# Supplementary figures and images for: Maternal body mass index and oxytocin in augmentation of labour in nulliparous women: a prospective observational study
Source: BMJ Open. 2021 Mar 25;11(3):e044754. doi: 10.1136/bmjopen-2020-044754 (PMC8006853; doi:10.1136/bmjopen-2020-044754)

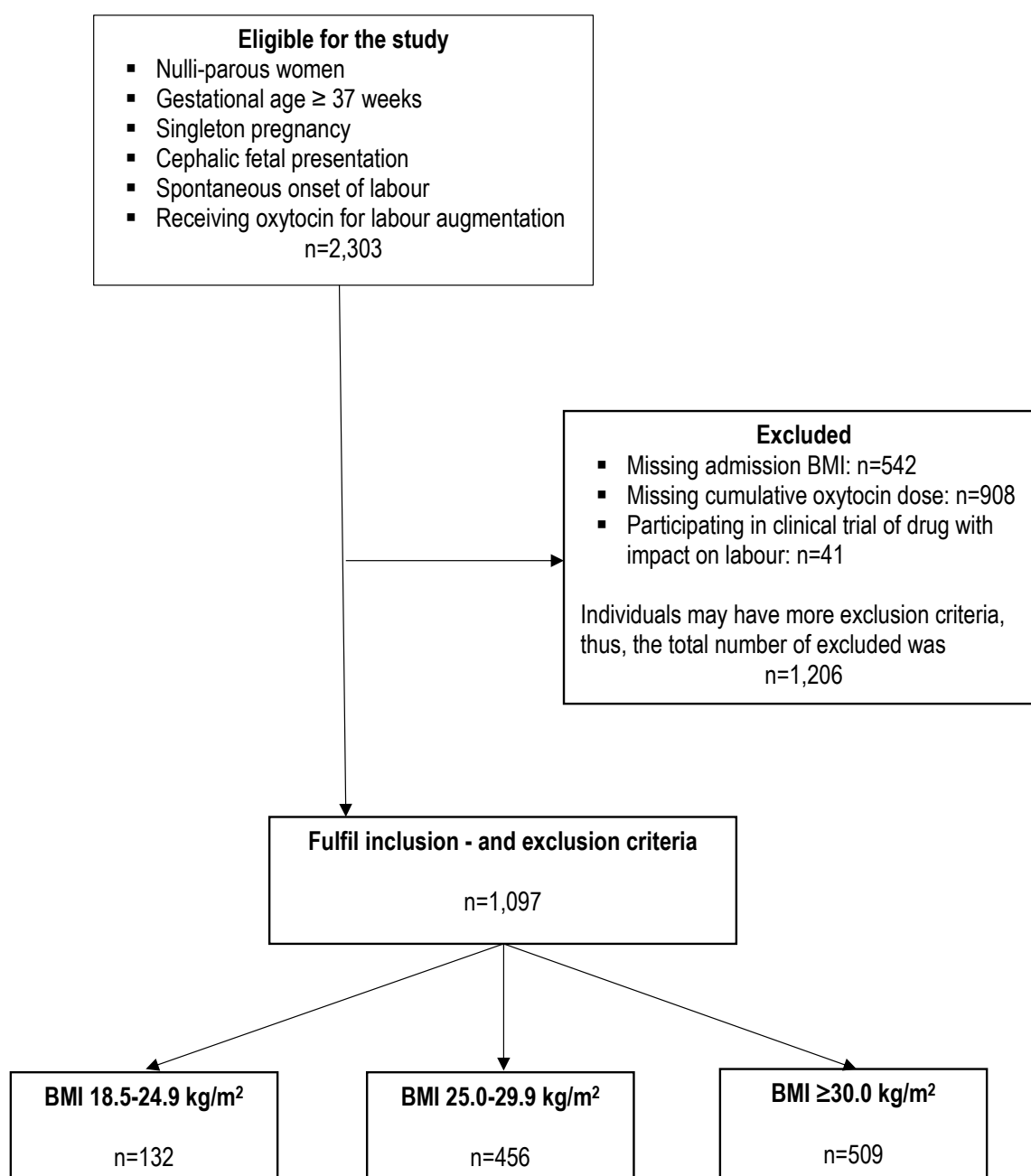

**Figure S1.** Flow chart of the study population

Supplement: Supplementary data [file bmjopen-2020-044754supp001.pdf]
