# Supplementary material for: Maternal body mass index and oxytocin in augmentation of labour in nulliparous women: a prospective observational study
Source: BMJ Open. 2021 Mar 25;11(3):e044754. doi: 10.1136/bmjopen-2020-044754 (PMC8006853; doi:10.1136/bmjopen-2020-044754)
Supplement: Supplementary data [file bmjopen-2020-044754supp002.pdf]

BMJ Publishing Group Limited (BMJ) disclaims all liability and responsibility arising from any reliance placed on this supplemental material which has been supplied by the author(s)

Table S1. Comparison of maternal, obstetric and neonatal characteristics in nulliparous women with singleton, term pregnancy with cephalic presentation, spontaneous start of labour and treatment with oxytocin infusion for labour augmentation included in the study and those excluded from the study.

	Included n=1,097	Excluded n=1,206	p-value
Maternal characteristics			
Maternal age (years)	28.5 [4.3]	28.5 [4.6]	NS*
Smoking during pregnancy (Yes/No)	35 / 1022 (3.3 / 96.7)	34 / 1091 (3.0 / 97.0)	NS†
Diabetes Mellitus (Yes/No)	12 / 2085 (1.1 / 98.9)	21 / 1185 (1.7 / 98.3)	NS†
Hypertension disorder (including Preeclampsia) (Yes/No)	31/1066 (2.8/97.2)	40 / 1166 (3.3 / 96.7)	NS†
Maternal weight in first trimester (kg)	68.9 [14.1]	68.1 [13.6], n=1,144	NS*
Gestational weight gain (kg)	15.1 [5.8]	14.8 [5.9], n=675	NS*
BMI in first trimester (kg/m^2)	24.8 [4.7]	24.7 [4.6], n=1,139	NS*
BMI at admission in labour (kg/m^2)	30.3 [5.0]	30.3 [5.0], n=664	NS*
Obstetric characteristics			
Gestational age at delivery (days)	282 [7.6]	282 [7.7]	NS*
Mode of delivery:			
Normal vaginal delivery	891 (81.2)	924 (76.6)	<0.01‡
Instrumental vaginal delivery	142 (12.9)	176 (14.6)	
Caesarean section (CS)	64 (5.8)	106 (8.8)	
Obstetric anal sphincter injury (Yes/No)	58 / 1039 (5.3 / 94.7)	49 / 1157 (4.1 / 95.9)	NS†
Indication CS (asphyxia/dystocia)	18 / 46 (28.1 / 71.9)	41 / 65 (38.7 / 61.3)	NS†
Epidural analgesia (Yes/No)	866 / 231 (78.9 / 21.1)	918 / 288 (76.1 / 23.9)	NS†
Postpartum haemorrhage (mL)	470 [368]	513 [406]	<0.001#
Postpartum haemorrhage >1000mL (Yes/No)	81 / 1008 (7.4 / 92.6)	98 / 866 (10.2 / 89.8)	0.03†
Neonatal characteristics			
Fetal birth weight (gram)	3560 [449]	3545 [466]	NS*
Apgar<7 at 5 minutes (Yes/No)	19 / 1078 (1.7 / 98.3)	32 / 1174 /2.7 / 97.3)	NS†
Apgar <4 at 5 minutes (Yes/No)	1 / 1096 (0.1 / 99.9)	4 / 1202 (0.3 / 99.7)	NS†
Umbilical arterial pH <7.0 (Yes/No)	14 / 1083 (1.3 / 98.7)	17 / 1189 (1.4 / 98.6)	NS†

Figures denote mean and [one standard deviation] or number and (%).
*One-way ANOVA; #Mann-Whitney U test; † Pearson's Chi-squared test (df=1). ‡ Pearson's Chi-squared test (df=2).
Level of significance p<0.05, NS= Not Significant
