# Supplementary material for: Maternal body mass index and oxytocin in augmentation of labour in nulliparous women: a prospective observational study
Source: BMJ Open. 2021 Mar 25;11(3):e044754. doi: 10.1136/bmjopen-2020-044754 (PMC8006853; doi:10.1136/bmjopen-2020-044754)
Supplement: Author's manuscript [file bmjopen-2020-044754.draft_revisions.pdf]

BMJ Open is committed to open peer review. As part of this commitment we make the peer review history of every article we publish publicly available.

When an article is published we post the peer reviewers' comments and the authors' responses online. We also post the versions of the paper that were used during peer review. These are the versions that the peer review comments apply to.

The versions of the paper that follow are the versions that were submitted during the peer review process. They are not the versions of record or the final published versions. They should not be cited or distributed as the published version of this manuscript.

BMJ Open is an open access journal and the full, final, typeset and author-corrected version of record of the manuscript is available on our site with no access controls, subscription charges or pay-per-view fees (http://bmjopen.bmj.com).

If you have any questions on BMJ Open's open peer review process please email info.bmjopen@bmj.com

BMJ Open

Maternal body mass index and oxytocin in augmentation of labour in nulliparous women: a prospective observational study

Journal:	*BMJ Open*
Manuscript ID	bmjopen-2020-044754
Article Type:	Original research
Date Submitted by the Author:	13-Sep-2020
Complete List of Authors:	Ramö Isgren, Anna; Linköping University, Department of Obstetrics and Gynaecology, Department of Biomedical and Clinical Sciences, University Hospital, Linköping<br>Kjolhede, Preben; Linköping University, Departement of Obstetrics and Gynaecology, Department of Biomedical and Clinical Sciences, University Hospital, Linköping<br>Carlhäll, Sara; Linköping University, Departement of Obstetrics and Gynaecology, Department of Biomedical and Clinical Sciences, University Hospital, Linköping<br>Blomberg, Marie; Linköping University, Department of Obstetrics and Gynaecology, Department of Biomedical and Clinical Sciences, University Hospital, Linköping
Keywords:	Maternal medicine < OBSTETRICS, Prenatal diagnosis < OBSTETRICS, Fetal medicine < OBSTETRICS

SCHOLARONE™
Manuscripts

I, the Submitting Author has the right to grant and does grant on behalf of all authors of the Work (as defined in the below author licence), an exclusive licence and/or a non-exclusive licence for contributions from authors who are: i) UK Crown employees; ii) where BMJ has agreed a CC-BY licence shall apply, and/or iii) in accordance with the terms applicable for US Federal Government officers or employees acting as part of their official duties; on a worldwide, perpetual, irrevocable, royalty-free basis to BMJ Publishing Group Ltd ("BMJ") its licensees and where the relevant Journal is co-owned by BMJ to the co-owners of the Journal, to publish the Work in this journal and any other BMJ products and to exploit all rights, as set out in our licence.

The Submitting Author accepts and understands that any supply made under these terms is made by BMJ to the Submitting Author unless you are acting as an employee on behalf of your employer or a postgraduate student of an affiliated institution which is paying any applicable article publishing charge ("APC") for Open Access articles. Where the Submitting Author wishes to make the Work available on an Open Access basis (and intends to pay the relevant APC), the terms of reuse of such Open Access shall be governed by a Creative Commons licence – details of these licences and which Creative Commons licence will apply to this Work are set out in our licence referred to above.

Other than as permitted in any relevant BMJ Author's Self Archiving Policies, I confirm this Work has not been accepted for publication elsewhere, is not being considered for publication elsewhere and does not duplicate material already published. I confirm all authors consent to publication of this Work and authorise the granting of this licence.

For peer review only

Maternal body mass index and oxytocin in augmentation of labour in nulliparous women: a prospective observational study

Anna Ramö Isgren, MD, Preben Kjølhede, MD, PhD, Sara Carlhäll, MD, PhD, Marie Blomberg, MD, PhD.

Affiliation

Department of Obstetrics and Gynaecology in Linköping, Department of Biomedical and Clinical Sciences, Linköping University, Sweden

Corresponding author

Anna Ramö Isgren, MD

Department of Obstetrics and Gynaecology

University Hospital

581 85 Linköping

Sweden

Telephone number: +46-706-365997

E-mail: Anna.Ramo.Isgren@regionostergotland.se

Word count: 3608

For peer review only - http://bmjopen.bmj.com/site/about/guidelines.xhtml

For peer review only

ABSTRACT

Objective To evaluate oxytocin use for augmentation of labour in relation to body mass index (BMI) at admission in labour, focusing on cumulative oxytocin dose and maximum rate of oxytocin infusion during the active phase of the first stage of labour.

Design Prospective observational study.

Setting Seven hospitals in Sweden.

Participants 1,097 nulliparous women with singleton cephalic presentation pregnancy, ≥37 weeks of gestation, spontaneous onset of labour and treatment with oxytocin infusion for labour augmentation. The study population was classified into three admission in labour BMI subgroups: normal weight (18.5-24.9), overweight (25.0-29.9), obese (≥30.0). The cumulative oxytocin dose was measured from the start of oxytocin infusion until the neonate was born.

Primary outcome Cumulative oxytocin dose.

Secondary outcome Maximum rate of oxytocin infusion during the active phase of first stage of labour.

Results The mean cumulative oxytocin dose increased in the BMI groups (normal weight 2278 mU, overweight 3108 mU and obese 4082 mU ($p<0.0001$)). However, when adjusted for the confounders (cervical dilatation when oxytocin infusion was started, fetal birth weight and epidural analgesia) the significant difference was no longer seen. The maximum oxytocin infusion rate during the first stage of labour differed significantly in the BMI groups. Together, but not individually, the confounding factors eliminated the statistical significance. In addition, the maximum oxytocin infusion rate in first stage of labour was significantly higher in women with emergency CS compared with women with vaginal delivery.

Conclusions Women with increasing BMI with augmentation of labour received a higher cumulative oxytocin dose and had a higher maximum oxytocin infusion rate during first stage of labour, however, when adjusted for relevant confounders the difference was no longer seen. In the

For peer review only - http://bmjopen.bmj.com/site/about/guidelines.xhtml

future, the guidelines for augmentation of labour with oxytocin infusion might be reconsidered and include modifications related to BMI.

Strengths and limitations of this study

- This is a large, prospective, multicentre study including six hospitals in Sweden.
- The study population was restricted to nulliparous women in full term pregnancy with spontaneous onset of labour.
- Detailed background characteristics of the study population included pregnancy, obstetric and neonatal characteristics.
- The national Swedish guidelines for the definition of spontaneous start of labour was used which may limit the generalisability of the study.

For peer review only

For peer review only - http://bmjopen.bmj.com/site/about/guidelines.xhtml

INTRODUCTION

Maternal obesity is associated with increased risks for obstetric complications.[1-4] In the first stage of labour, obese women have been reported to have a slower progression with increased risks for dystocia which, in turn, are risk factors for chorioamnionitis, emergency Caesarean section (CS) and major postpartum bleeding.[4-13] With a higher incidence of dystocia women with obesity have an increased need for treatment with oxytocin infusion for augmentation during labour. Previous studies have indicated that women with higher body mass index (BMI) receive larger oxytocin infusion doses and higher rates of oxytocin infusion in augmentation and induction of labour.[14-18] However, there is heterogeneity in the design of these studies and the results are not entirely consistent. Today, the guidelines for oxytocin administration in augmentation of labour differ worldwide and none of the guidelines adjust for maternal weight or BMI.[19-21] More research is needed to establish evidence-based clinical guidelines for the use of oxytocin during labour in relation to BMI.

This study evaluates oxytocin use for augmentation of labour in nulliparous women with spontaneous onset at term in relation to maternal admission at labour BMI. The primary aim was to determine the cumulative oxytocin dose in relation to the woman´s BMI. Secondary aim was to analyse the association between maternal BMI and maximum rate of oxytocin infusion during the active phase of the first stage of labour.

For peer review only - http://bmjopen.bmj.com/site/about/guidelines.xhtml

METHODS

This is a prospective observational multicentre study conducted between April 2017 and February 2019 in all seven departments of Obstetrics and Gynaecology in the southeast health region of Sweden.

Inclusion criteria were nulliparous women with a singleton cephalic presentation pregnancy, ≥37 weeks of gestation with spontaneous onset of labour (i.e. group one in The Ten Group Classification System[22]), and known admission BMI, who were treated with oxytocin infusion for labour augmentation and the cumulative oxytocin dose was measured.

All participating maternity wards adhered to the same national clinical guidelines concerning the definition of spontaneous onset of labour and for augmentation with oxytocin during active labour.[19 23] Likewise, the wards had identical protocols for oxytocin augmentation. The Swedish recommendation for the definition of active spontaneous onset of labour states that at least two out of three of the following criteria have to be fulfilled: spontaneous rupture of the membrane, regular painful contractions (2-3/10 minutes), and the cervix dilated four centimetres or effaced and dilated more than 1 centimetre. In addition to these criteria, the labour should progress within the following two hours.[23] The recommendation for initiation of augmentation with oxytocin during the active phase of the first stage of labour was defined as delayed progress of three hours from the expected 1 cm/hour cervical dilatation and during the second stage defined as no descent for one hour, or active pushing for more than 30 minutes.[19] The guidelines for start of oxytocin infusion also entails continuously cardiotocography (CTG), for registration of the fetal heart rate and monitoring contractions of the uterus.

Oxytocin was administered as an intravenous infusion through a peripheral venous catheter by an electronic infusion pump (B BRAUN Infusomat® Space or Alaris™ GP Plus). A standard solution for infusion of oxytocin for augmentation during labour was prepared by adding 1 ml (containing 5 units of oxytocin) to 500 ml of 0.9% sodium chloride, reaching a concentration of 10

For peer review only - http://bmjopen.bmj.com/site/about/guidelines.xhtml

mU/ml. The infusion was routinely started at an infusion rate of 3.3 mU/min and the rate was increased every 20 minutes by 3.3 mU/min at a time until 4-5 contractions/10 minutes were achieved and/or normal progress of labour occurred according to the partograph, a graphic record of the progress of labour. Hyperstimulation was defined as >5 contractions/10 minutes.[19] The cumulative oxytocin dose was automatically registered by the infusion pump and was measured from the start of the oxytocin infusion until the neonate was born. The attending midwife registered the rate of oxytocin infusion manually in the partograph continuously during labour, and took the reading from the pump after delivery for registration of the cumulative oxytocin dose.

Maternal height was reported or measured at the first antenatal visit and maternal weight was measured at admission to the labour ward, which enabled calculation of admission BMI (kg/m^2). The study population was classified into three subgroups according to the World Health Organization (WHO) BMI classification based on their BMI at the time of admission to the labour ward: normal weight (BMI 18.5-24.9), overweight (BMI 25.0-29.9), and obese (BMI ≥30.0).

Demographic and clinical data comprising maternal, obstetric and neonatal characteristics were prospectively recorded in standardised medical records (Obstetrix, Cerner). The maternal characteristics consisted of age, smoking habit, diabetes mellitus, hypertension disorder (including preeclampsia), and maternal weight in the first trimester and at delivery. The obstetric characteristics included gestational length at delivery, epidural analgesia, mode of delivery, occurrence of obstetric anal sphincter injury, and postpartum haemorrhage. In addition, the obstetric characteristics included cervical dilatation at the start of oxytocin augmentation subdivided into three categories: < 6 cm, 6-10 cm and fully dilated,[24] the time from the start of active labour until the start of oxytocin augmentation, the time from the start of oxytocin augmentation until delivery, the cumulative oxytocin dose from the start of oxytocin infusion to delivery, and the maximum oxytocin infusion rate during the first stage of active labour.

The neonatal characteristics comprised fetal birth weight, Apgar score (<7 and <4 at 5 minutes) and umbilical arterial pH <7.0.

For peer review only - http://bmjopen.bmj.com/site/about/guidelines.xhtml

For peer review only

Statistics

Sample size estimation was based on the cumulative oxytocin dose, given the assumptions that the minimal clinically important difference in cumulative oxytocin dose, between groups was 1200 mU, and that the standard deviation of the cumulative oxytocin dose was 3000 mU. Thus, in order to detect a statistically significant difference at a 5% level with 80% power the sample size was estimated to be approximately 100 in each group.

Data are presented as mean and one standard deviation (SD), or number and per cent. Comparisons of nominal data were conducted by means of Pearson Chi-squared tests. Continuous data were compared using one-way analysis of variance (ANOVA) tests for normally distributed data and Mann-Whitney U-tests for non-normally distributed data in the univariate analyses. The outcome measures were evaluated in multivariate analyses by means of two-way ANOVA or analysis of covariance (ANCOVA), adjusting for the confounding factors cervical dilation at start of oxytocin infusion, categorised in < 6 cm, 6-10 cm, and fully dilated, fetal birth weight (as a continuous variable) and use of epidural analgesia (dichotomised in yes or no). In the multivariate analyses, the non-normally distributed continuous data variables underwent logarithmic transformation. Post-hoc tests were performed by means of Tukey's honestly significant difference (HSD) test. The level of significance was set at $p<0.05$ for two-tailed tests. Statistical analyses were performed using the software TIBCO Statistica™, version 13.5 (TIBCO Software Inc, Palo Alto, CA 94303, USA).

Patient and public involvement

Patients or the public were not involved in the design, or conduct, or reporting or dissemination plans of our research.

For peer review only - http://bmjopen.bmj.com/site/about/guidelines.xhtml

RESULTS

The selection of the study population is presented in the flow chart (online Supplementary Figure S1). A total of 1,097 women were included in the study. Maternal, obstetric and neonatal characteristics of the study population in relation to BMI group are presented in table 1.

For peer review only

For peer review only - http://bmjopen.bmj.com/site/about/guidelines.xhtml

Table 1. Maternal, obstetric and neonatal characteristics in nulliparous women with singleton, term pregnancy with cephalic presentation and augmentation of labour with oxytocin infusion according to admission in labour body mass index.

	BMI 18.5-24.9 (n= 132)	BMI 25.0-29.9 (n=456)	BMI ≥30.0 (n=509)
Maternal characteristics			
Maternal age (years)	28.1 [4.1]	28.8 [4.4]	28.2[4.2]
Smoking during pregnancy	5 (3.8)	11 (2.4)	19 (3.7)
Diabetes mellitus	0 (0)	4 (0.9)	8 (1.6)
Hypertension disorder (including preeclampsia)	0 (0)	7 (1.5)	24 (4.7)
Maternal weight in first trimester (kg)	56.1 [6.1]	62.4 [6.7]	78.3 [14.4]
Gestational weight gain (kg)	11.1 [3.6]	14.2 [4.4]	16.9 [6.6]
Obstetric characteristics			
Gestational age at delivery (days)	281.4 [7.8]	281.5 [7.7]	282.4 [7.5]
Time from active labour until start of oxytocin (h)	8.6 [4.0] (n=105)	9.0 [5.3] (n=385)	8.4 [4.6] (n=422)
Time from start of oxytocin until delivery (h)	3.0 [2.7]	3.8 [3.4]	5.0 [4.1]
Cervical dilatation at start of oxytocin: < 6 cm	16 (12.1)	87 (19.1)	144 (28.3)
6-10 cm	34 (25.8)	142 (31.1)	207 (40.7)
Fully dilated	82 (62.1)	227 (49.8)	158 (31.0)
Mode of delivery: Normal vaginal delivery	107 (81.1)	379 (83.1)	405 (79.6)
Instrumental vaginal delivery	22 (16.7)	59 (12.9)	61 (12.0)
Caesarean section	3 (2.3)	18 (3.9)	43 (8.4)
Obstetric anal sphincter injury	8 (6.1)	22 (4.8)	28 (5.5)
Postpartum haemorrhage (mL)	456 [350]	454 [378]	488 [363]
Postpartum haemorrhage > 1000 mL	10 (7.6)	31 (6.9)	40 (7.9)
Epidural analgesia (no. of women)	96 (72.7)	355 (77.9)	415 (81.5)
Neonatal characteristics			
Fetal birth weight (gram)	3329 [351]	3515 [420]	3661 [467]
Apgar score <7 at 5 minutes (no. of infants)	1 (0.8)	7 (1.5)	11 (2.2)
Apgar score <4 at 5 minutes (no. of infants)	0 (0)	0 (0)	1 (0.2)
Umbilical arterial pH <7.0 (no. of infants)	0 (0) (n=110)	7 (1.5) (n=393)	7 (1.4) (n=442)

Figures denote mean and [one standard deviation] or number and (%)

For peer review only

For peer review only - http://bmjopen.bmj.com/site/about/guidelines.xhtml

As shown in table 2, the cumulative oxytocin dose increased significantly with increasing BMI group (normal weight, mean 2278 mU [2748 mU]; overweight, mean 3108 mU [3839 mU], and obese, mean 4082 mU [4895 mU]; One-way ANOVA $p<0.0001$). However, when adjusting for all three confounders, the significance vanished between the three BMI groups (Table 2).

For peer review only

For peer review only - http://bmjopen.bmj.com/site/about/guidelines.xhtml

210 **Table 2.** Cumulative oxytocin dose and maximum rate of oxytocin during active phase of first stage of labour according to body mass index.

	BMI 18.5-24.9	BMI 25.0-29.5	BMI ≥30	ANOVA / ANCOVA between BMI groups, p-value				
				Crude	Adjusted [1]	Adjusted [2]	Adjusted [3]	Adjusted [4]
	(n=132)	(n=456)	(n=509)					
Cumulative oxytocin dose (mU)	2278 [2748]	3108 [3839]	4082 [4895]	<0.0001	0.42	<0.001	<0.0001	0.88
Independent predictive factors:								
Cervical dilatation					<0.0001			<0.0001
Fetal birth weight						<0.0001		<0.0001
Epidural analgesia							<0.0001	<0.0001
	(n=49)	(n=220)	(n=347)					
Maximum rate of oxytocin during active phase of first stage of labour (mU/min)	12.7 [7.1]	13.6 [9.1]	15.5 [9.5]	<0.01	<0.05	<0.05	0.01	0.10
Independent predictive factor:								
Cervical dilatation					<0.0001			<0.0001
Fetal birth weight						<0.01		<0.0001
Epidural analgesia							<0.01	<0.01

211 Figures denote mean and [SD]

212 1 Adjusted for cervical dilatation at start of oxytocin infusion

213 2 Adjusted for fetal birth weight

214 3 Adjusted for epidural analgesia

215 4 Adjusted simultaneously for 1, 2, and 3.

216

217

218

219

220

For peer review only - http://bmjopen.bmj.com/site/about/guidelines.xhtml

Each of the three confounders were strong independent predictors of the cumulative oxytocin dose, but adjusted simultaneously together they equalise the differences in cumulative oxytocin dose completely between the BMI groups (Figure 1).

The maximum rate of oxytocin infusion during the active phase of the first stage of labour differed significantly in the BMI groups in the univariate analyses (normal weight (n=49), mean 12.7 mU/min [7.1 mU/min], overweight (n=220), mean 13.6 mU/min [9.1 mU/min], and obese (n=347), mean 15.5 mU/min [9.5 mU/min]; One-way ANOVA, p<0.01). Individually, the confounding factors did not influence this, but when the analysis was conducted with all three confounding factors simultaneously, the significant difference between the BMI groups disappeared (Table 2, Figure 2).

When analysing the subgroup of women with overweight and obesity and known maximum rate of oxytocin infusion during the active phase of the first stage of labour, the maximum infusion rate was significantly associated with mode of delivery in both BMI groups when adjusted simultaneously for cervical dilation, fetal birth weight and epidural analgesia (Figures 3, 4).

The time from active labour until the start of oxytocin infusion did not differ between the BMI groups (One-way ANOVA; p=0.27), (Table 1), even when adjusted for fetal birth weight and epidural analgesia (ANCOVA, p=0.18). A significantly higher proportion of women with obesity ended with an emergency CS compared with normal weight and overweight women (8.4% versus 2.3% and 3.9%, respectively) (Pearson Chi-squared test; df=2, p<0.01) whereas no significant differences were seen between the BMI groups in women with a normal vaginal delivery or instrumental vaginal delivery.

The analysis of the women included in the study group versus those who were excluded mainly because of missing cumulative oxytocin dose showed similar proportions of characteristics, including indication for CS, in the two groups except for the rate of CS (5.8% in the study group versus 8.8% in the group of excluded women) and the occurrence of postpartum haemorrhage exceeding 1000 mL (7.4% versus 10.2%) (Online Supplementary Table S1). Further analysis was

For peer review only - http://bmjopen.bmj.com/site/about/guidelines.xhtml

conducted between the women who had CS with registration of cumulative oxytocin dose (n=64) and those women who had CS but oxytocin dose was not registered (n=106). The degree of cervical dilation when oxytocin augmentation started did not differ nor did the time from the start of active labour to the start of infusion of oxytocin differ (data not shown). The time from the start of oxytocin augmentation to delivery was significantly shorter in those women where the cumulative oxytocin dose was not registered (mean 4.1 h (3.6 h) versus 8.6 h (5.1 h) (Mann-Whitney U-test, $p<0.01$)).

For peer review only

For peer review only - http://bmjopen.bmj.com/site/about/guidelines.xhtml

DISCUSSION

Principal findings

The present study showed that nulliparous women at term with spontaneous onset and augmentation of labour in unadjusted analysis received a higher cumulative oxytocin dose and had a higher maximum oxytocin infusion rate at first stage of labour with increasing maternal admission BMI. After adjusting for cervical dilation at start of oxytocin infusion, fetal birth weight and epidural analgesia, the significant difference was no longer seen. In addition, the maximum oxytocin infusion rate in first stage of labour was significantly higher in women with emergency CS compared with vaginal delivery.

Strengths and limitations

The major strength of the study is the large number of participants restricted to nulliparous women with spontaneous onset of labour. The multicentre design made it possible to include women with different sociodemographic backgrounds, including both urban and rural areas. In addition, the labour wards included in the study had the same clinical guidelines for obstetric care.

The study has some limitations. Since there are no known cut-off values for BMI during pregnancy we used the definition according to the WHO criteria. There are some limitations with the use of admission in labour BMI. Except increased fat mass, increased volume of extravascular fluid and blood, increased weight from maternal tissue from breast and uterus, admission BMI also includes the weight of the fetus, placenta and amniotic fluid. However, these limitations do not affect the purpose of this study, which was to find a relationship between BMI at admission to the labour ward, and the cumulative oxytocin dose and maximum oxytocin infusion rate in the first stage of labour.

There might be a risk of selection bias since the cumulative oxytocin dose was not registered for all women. However, in order to determine the potential scope of selection bias we compared

For peer review only

For peer review only - http://bmjopen.bmj.com/site/about/guidelines.xhtml

the study group with the women that were excluded from the study. The data showed that the groups were similar except for the proportions of CS and postpartum haemorrhage, which were much higher in the excluded group. A plausible explanation for this discrepancy might be the time pressure that the staff were exposed to when a delivery was converted to an emergency CS or heavy postpartum bleeding occurred, and thus they forgot to record the cumulative oxytocin dose. All these aspects speak against selection bias.

Another limitation is our definition of the start of active labour. We used the national Swedish guidelines for the definition of spontaneous start of labour. There is no consensus worldwide either of the definition of spontaneous start of active labour or the definition of dystocia and this may limit the generalizability of the study. All participating sites in this study have well established routines if hyperstimulation occurs (e.g. reduce or stop the oxytocin infusion); unfortunately, we did not have the exact number of periods of hyperstimulation registered as the CTG-traces were not scrutinised in the study.

Interpretation in relation to other studies

This prospective study showed that women with increasing BMI received a higher cumulative dose of oxytocin compared with normal weight women, however, when adjusting for cervical dilation at start of oxytocin infusion no significant difference between the BMI groups were seen. Soni et al also studied women with spontaneous start of labour and treatment with oxytocin for arrest of dilation. They found that the total amount of oxytocin administrated was significantly higher in a group of women with admission BMI > 35 (n=23) compared with women in three lower BMI groups. The BMI-groups did not differ according to EDA or birth weight, however, the cervical dilatation at arrest differed significantly and were not adjusted for in the study.[14]

The present study showed that women with increasing BMI received a higher maximum infusion rate of oxytocin for augmentation in the first stage of labour even after adjustment for cervical dilation at start of oxytocin infusion. When additionally adjusting for fetal birth weight and epidural

For peer review only - http://bmjopen.bmj.com/site/about/guidelines.xhtml

analgesia the difference were no longer seen. The maximum oxytocin infusion rate in the first stage of labour in relation to maternal BMI has not been studied before in women with spontaneous start of labour. However, Hill et al. showed in induced labour that obese women (BMI >40) had a significantly higher mean maximum oxytocin infusion rate during first stage of labour compared with a group of lean women (BMI<28), matched for gestational age, fetal birth weight and cervical dilatation and fetal station at admission.[15] Adam´s et al studied oxytocin use in women with induced and spontaneous onset of labour and demonstrated that women with class III obesity had an increased risk for an oxytocin rate >20 mU/min, from admission until vaginal delivery, compared with lower BMI groups. However, in a sub analysis on spontaneous onset of labour, no significance was seen.[18]

The present study showed that women with obesity started oxytocin infusion at an earlier cervical stage compared with lower BMI groups. The reason for this can be discussed. Earlier studies have showed a longer first, but not second, stage of labour in obese women.[4 8] The time point when the oxytocin infusion starts equals when labour dystocia is diagnosed. Obese women receive oxytocin infusion at an earlier cervical dilatation stage which could indicate an abnormal uterine contractility. Underlying pathophysiological mechanisms and ineffective uterine contractility among obese women have been studied. From biopsies, Zhang et al showed that the myometrium from obese women undergoing elective CS at term contracted with less frequency and force compared with the myometrium from normal weight women.[6] Other studies have indicated a different metabolic profile in obese women that could affect the contraction strength in the myometrium in an inhibitory way.[25 26]

Clinical implications

There is evidence that oxytocin administration increases the risk for uterine hyperstimulation during labour, which can lead to fetal heart rate abnormalities. Furthermore, observational studies have shown an association between oxytocin use and increased risk for postpartum haemorrhage.[27]

For peer review only - http://bmjopen.bmj.com/site/about/guidelines.xhtml

Therefore, clinicians must use oxytocin with caution with respect to the potential risks and simultaneously be aware of the consequence of a CS due to dystocia and insufficient treatment with oxytocin in obese women. Our study showed that the maximum oxytocin infusion rate was significantly increased in overweight and obese women with emergency CS compared with vaginal deliveries. The reason for this is unclear but the explanation could be labour dystocia that did not respond to oxytocin treatment, or fetal distress due to uterine hyperstimulation. Further research is needed to explore why women respond differently to oxytocin treatment. Perhaps there is a different bodily distribution of administrated oxytocin in different BMI groups.

Conclusion and future directions

This large prospective study showed that women with increasing BMI received a higher cumulative oxytocin dose and higher maximum oxytocin infusion rate during the active phase of first stage of labour, however, when adjusting for cervical dilation at start of oxytocin infusion, fetal birth weight and use of epidural analgesia, the significant difference disappeared.

Oxytocin is one of the most commonly used drugs in the maternity wards and obesity is an increasing and major concern in obstetric healthcare. There are still knowledge gaps and further research is needed regarding the pathophysiological mechanisms in obese women affecting the dosage and duration of oxytocin in labour augmentation. In order to optimise treatment of labour dystocia, the patient care has to be individualized and the guidelines for augmentation of labour with oxytocin infusion might in the future be reconsidered and include modifications related to maternal BMI.

For peer review only - http://bmjopen.bmj.com/site/about/guidelines.xhtml

Acknowledgements

We would like to thank the midwives and staff of the participating hospitals for collecting data.

Contribution to Authorship

All authors conceived the study idea, interpreted the data analysis and critically revised the manuscript. ARI acquired the data and PK performed the data analysis. All the authors agree to take responsibility for the work.

Funding

This work was supported by the Medical Research Council of Southeast Sweden, FORSS (FORSS-756621).Unrestricted grants were obtained from the County Council of Östergötland and Linköping University, Sweden. The funders were not involved in the development of the study from idea to project plan, implementation, analysis of data or writing of the manuscript.

Competing interests

The authors have no conflicts of interest to disclose.

Patient consent for publication

Not required

Details of Ethical Approval

As required by Swedish law and regulations the study was approved by the Ethical Review Board at Linköping University (Dnr 2017/277-31).

Data availability statement

Data are available upon reasonable request and according to Swedish legislation. Data statement section, technical appendix, statistical code and dataset available from the authors.

For peer review only

REFERENCES

1. Cedergren MI. Maternal morbid obesity and the risk of adverse pregnancy outcome. *Obstet Gynecol* 2004;103(2):219-24. doi: 10.1097/01.AOG.0000107291.46159.00
2. Baeten JM, Bukusi EA, Lambe M. Pregnancy complications and outcomes among overweight and obese nulliparous women. *American journal of public health* 2001;91(3):436-40. doi: 10.2105/ajph.91.3.436 [published Online First: 2001/03/10]
3. Blomberg M. Maternal obesity and risk of postpartum hemorrhage. *Obstet Gynecol* 2011;118(3):561-8. doi: 10.1097/AOG.0b013e31822a6c59 [published Online First: 2011/08/24]
4. Carlhall S, Kallen K, Blomberg M. Maternal body mass index and duration of labor. *European journal of obstetrics, gynecology, and reproductive biology* 2013;171(1):49-53. doi: 10.1016/j.ejogrb.2013.08.021 [published Online First: 2013/09/18]
5. Hilliard AM, Chauhan SP, Zhao Y, et al. Effect of obesity on length of labor in nulliparous women. *American journal of perinatology* 2012;29(2):127-32. doi: 10.1055/s-0031-1295653 [published Online First: 2011/11/23]
6. Zhang J, Bricker L, Wray S, et al. Poor uterine contractility in obese women. *BJOG : an international journal of obstetrics and gynaecology* 2007;114(3):343-8. doi: 10.1111/j.1471-0528.2006.01233.x [published Online First: 2007/01/31]
7. Kominiarek MA, Zhang J, Vanveldhuisen P, et al. Contemporary labor patterns: the impact of maternal body mass index. *Am J Obstet Gynecol* 2011;205(3):244 e1-8. doi: 10.1016/j.ajog.2011.06.014
8. Vahratian A, Zhang J, Troendle JF, et al. Maternal prepregnancy overweight and obesity and the pattern of labor progression in term nulliparous women. *Obstet Gynecol* 2004;104(5 Pt 1):943-51. doi: 10.1097/01.aog.0000142713.53197.91 [published Online First: 2004/11/02]
9. Norman SM, Tuuli MG, Odibo AO, et al. The effects of obesity on the first stage of labor. *Obstet Gynecol* 2012;120(1):130-5. doi: 10.1097/AOG.0b013e318259589c [published Online First: 2012/08/24]
10. Verdiales M, Pacheco C, Cohen WR. The effect of maternal obesity on the course of labor. *Journal of perinatal medicine* 2009;37(6):651-5. doi: 10.1515/jpm.2009.110 [published Online First: 2009/07/14]
11. Cedergren MI. Non-elective caesarean delivery due to ineffective uterine contractility or due to obstructed labour in relation to maternal body mass index. *European journal of obstetrics, gynecology, and reproductive biology* 2009;145(2):163-6. doi: 10.1016/j.ejogrb.2009.05.022 [published Online First: 2009/06/16]
12. Cheng YW, Shaffer BL, Bryant AS, et al. Length of the first stage of labor and associated perinatal outcomes in nulliparous women. *Obstet Gynecol* 2010;116(5):1127-35. doi: 10.1097/AOG.0b013e3181f5eaf0 [published Online First: 2010/10/23]
13. Nyflot LT, Stray-Pedersen B, Forsen L, et al. Duration of labor and the risk of severe postpartum hemorrhage: A case-control study. *PloS one* 2017;12(4):e0175306. doi: 10.1371/journal.pone.0175306 [published Online First: 2017/04/07]
14. Soni S, Chivan N, Cohen WR. Effect of maternal body mass index on oxytocin treatment for arrest of dilatation. *Journal of perinatal medicine* 2013;41(5):517-21. doi: 10.1515/jpm-2013-0024 [published Online First: 2013/03/22]
15. Hill M, Reed KL, Cohen WR. Oxytocin utilization for labor induction in obese and lean women. *Journal of perinatal medicine* 2015;43(6):703-6. doi: 10.1515/jpm-2014-0134 [published Online First: 2014/09/16]
16. Pevzner L, Powers BL, Rayburn WF, et al. Effects of maternal obesity on duration and outcomes of prostaglandin cervical ripening and labor induction. *Obstet Gynecol* 2009;114(6):1315-21. doi: 10.1097/AOG.0b013e3181bfb39f [published Online First: 2009/11/26]

For peer review only - http://bmjopen.bmj.com/site/about/guidelines.xhtml

17. Roloff K, Peng S, Sanchez-Ramos L, et al. Cumulative oxytocin dose during induction of labor according to maternal body mass index. *International journal of gynaecology and obstetrics: the official organ of the International Federation of Gynaecology and Obstetrics* 2015;131(1):54-8. doi: 10.1016/j.ijgo.2015.04.038 [published Online First: 2015/07/27]
18. Adams AD, Coviello EM, Drassinower D. The Effect of Maternal Obesity on Oxytocin Requirements to Achieve Vaginal Delivery. *American journal of perinatology* 2020;37(4):349-56. doi: 10.1055/s-0039-1694982 [published Online First: 2019/08/21]
19. Wibke J, Berg M, Dykes A-K, et al. National medical guidelines for the use of intrapartum oxytocin (Nationella medicinska Indikationer - Indikation för värkstimulering med oxytocin under aktiv förlossning). National Board of Health and Welfare. *Rapport number 2011:08 from the cooperational project "National medical guidelines" (från samarbetsprojektet Nationella medicinska Indikationer)* 2011;2011:1.
20. Page K, McCool WF, Guidera M. Examination of the Pharmacology of Oxytocin and Clinical Guidelines for Use in Labor. *Journal of midwifery & women's health* 2017;62(4):425-33. doi: 10.1111/jmwh.12610 [published Online First: 2017/07/14]
21. WHO Recommendations for Augmentation of Labour. Geneva: World Health Organization; 2014.
22. Robson M. The Ten Group Classification System (TGCS) - a common starting point for more detailed analysis. *BJOG : an international journal of obstetrics and gynaecology* 2015;122(5):701. doi: 10.1111/1471-0528.13267 [published Online First: 2015/01/21]
23. Definition of atctive labour. In Swedish: Definition av etablerat förlossningsarbete.2015 Swedish Association of Midwifes and Swedish Society of obstetrics and Gynecology.. Available at:https://www.barnmorskeförbundet.se/aktuellt/förbundet/definition-etablerat-forlossningsarbete/.[Accessed june 2020].
24. Zhang J, Landy HJ, Branch DW, et al. Contemporary patterns of spontaneous labor with normal neonatal outcomes. *Obstet Gynecol* 2010;116(6):1281-7. doi: 10.1097/AOG.0b013e3181fdef6e
25. Hehir MP, Morrison JJ. The adipokine apelin and human uterine contractility. *American journal of obstetrics and gynecology* 2012;206(4):359.e1-5. doi: 10.1016/j.ajog.2012.01.032 [published Online First: 2012/03/01]
26. Moynihan AT, Hehir MP, Glavey SV, et al. Inhibitory effect of leptin on human uterine contractility in vitro. *American journal of obstetrics and gynecology* 2006;195(2):504-9. doi: 10.1016/j.ajog.2006.01.106 [published Online First: 2006/05/02]
27. Rousseau A, Burguet A. Oxytocin administration during spontaneous labor: Guidelines for clinical practice. Chapter 5: Maternal risk and adverse effects of using oxytocin augmentation during spontaneous labor. *J Gynecol Obstet Hum Reprod* 2017;46(6):509-21. doi: 10.1016/j.jogoh.2017.04.009 [published Online First: 2017/05/06]

For peer review only - http://bmjopen.bmj.com/site/about/guidelines.xhtml

Figure legends

Figure 1.Cumulative oxytocin dose in nulliparous women with singleton, term, pregnancy with cephalic presentation and augmentation of labour according to admission body mass index.

Plots indicate mean and bars indicate 95% confidence interval. Adjusted for cervical dilatation at start of oxytocin infusion, fetal birth weight and epidural analgesia.

Figure 2. Maximum oxytocin infusion rate in first stage of labour in nulliparous women with singleton, term, pregnancy with cephalic presentation and augmentation of labour according to admission body mass index.

Plots indicate mean and bars indicate 95% confidence interval. Adjusted for cervical dilatation at start of oxytocin infusion, fetal birth weight and epidural analgesia.

Figure 3. Maximum oxytocin rate in active phase of first stage of labour and mode of delivery in overweight, nulliparous women with singleton, term pregnancy with cephalic presentation and augmentation of labour. Plots indicate mean and bars indicate 95% confidence interval. Adjusted for cervical dilatation at start of oxytocin infusion, fetal birth weight and epidural analgesia.

Figure 4. Maximum oxytocin rate in active phase of first stage of labour and mode of delivery in obese, nulliparous women with singleton, term pregnancy with cephalic presentation and augmentation of labour. Plots indicate mean and bars indicate 95% confidence interval. Adjusted for cervical dilatation at start of oxytocin infusion, fetal birth weight and epidural analgesia.

Legends to Supplementary Files

Supplementary Figure S1. Flow chart of the study population

Supplementary Table S1. Comparison of maternal, obstetric and neonatal characteristics in nulliparous women with singleton, term pregnancy with cephalic presentation, spontaneous start of

For peer review only

labour and treatment with oxytocin infusion for labour augmentation included in the study and those excluded from the study.

For peer review only

For peer review only - http://bmjopen.bmj.com/site/about/guidelines.xhtml

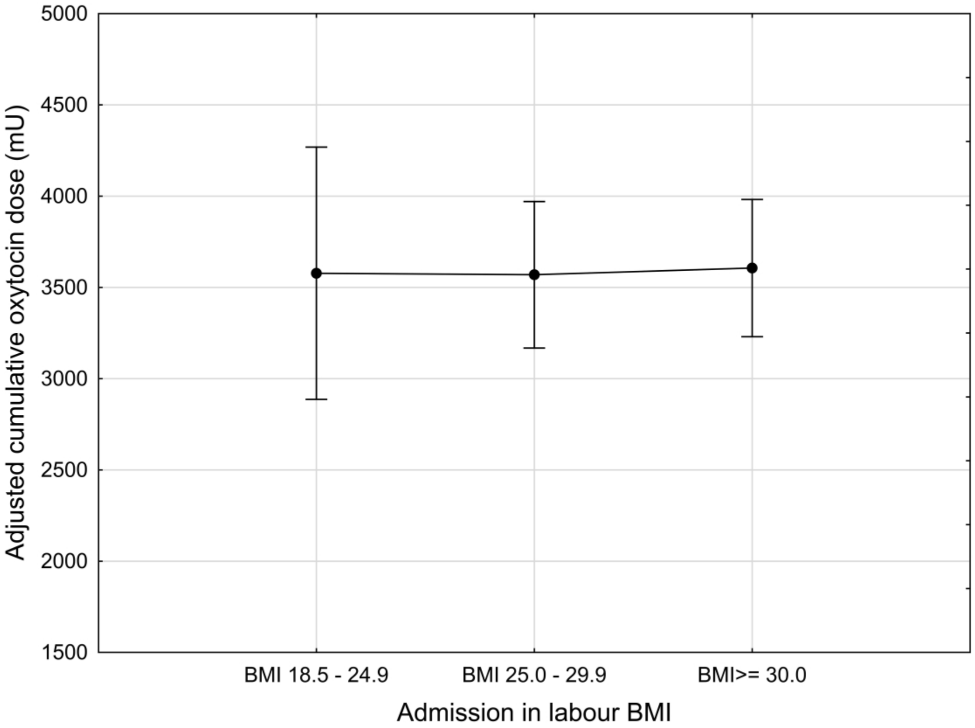

Figure 1.Cumulative oxytocin dose in nulliparous women with singleton, term, pregnancy with cephalic presentation and augmentation of labour according to admission body mass index. Plots indicate mean and bars indicate 95% confidence interval. Adjusted for cervical dilatation at start of oxytocin infusion, fetal birth weight and epidural analgesia.

1587x1190mm (96 x 96 DPI)

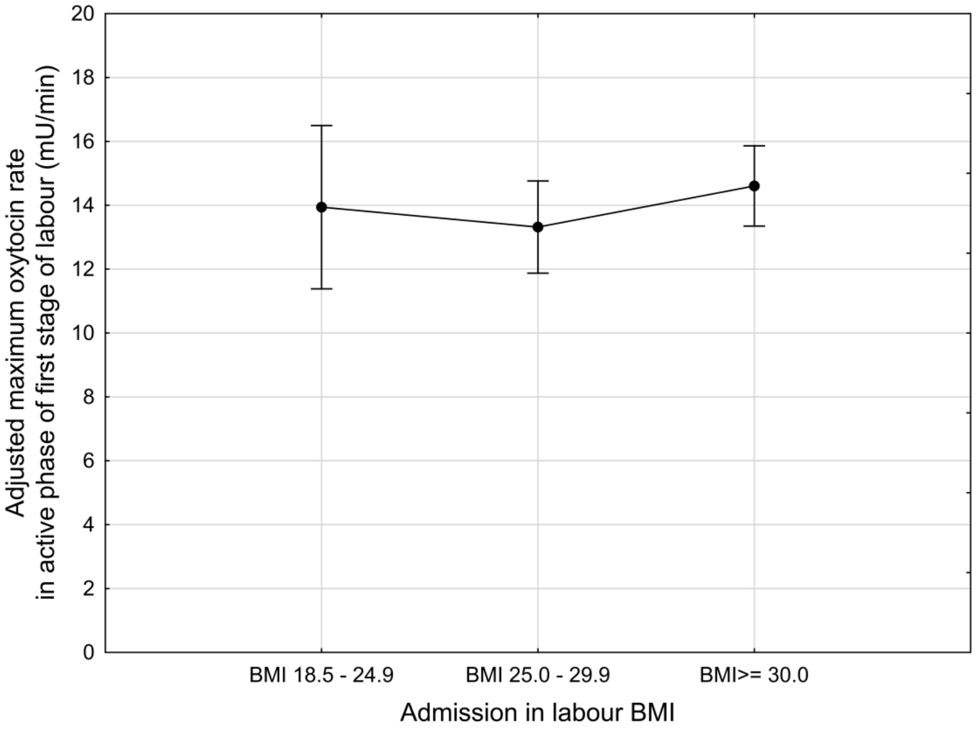

Figure 2. Maximum oxytocin infusion rate in first stage of labour in nulliparous women with singleton, term, pregnancy with cephalic presentation and augmentation of labour according to admission body mass index. Plots indicate mean and bars indicate 95% confidence interval. Adjusted for cervical dilatation at start of oxytocin infusion, fetal birth weight and epidural analgesia.

1587x1190mm (96 x 96 DPI)

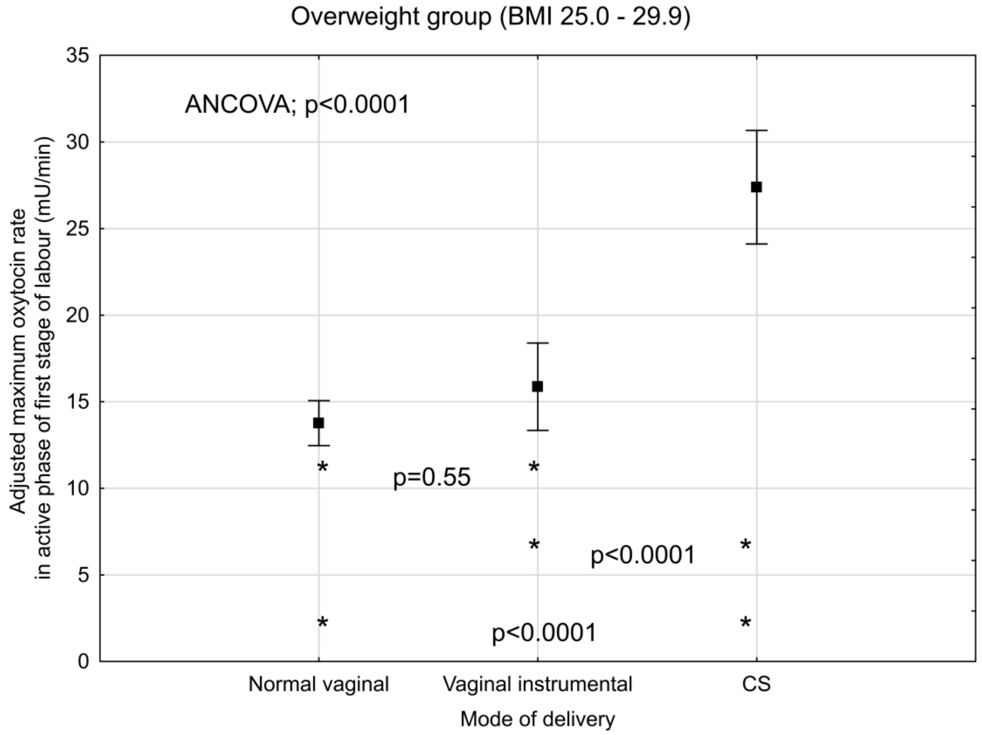

Figure 3. Maximum oxytocin rate in active phase of first stage of labour and mode of delivery in overweight, nulliparous women with singleton, term pregnancy with cephalic presentation and augmentation of labour. Plots indicate mean and bars indicate 95% confidence interval. Adjusted for cervical dilatation at start of oxytocin infusion, fetal birth weight and epidural analgesia

1587x1190mm (96 x 96 DPI)

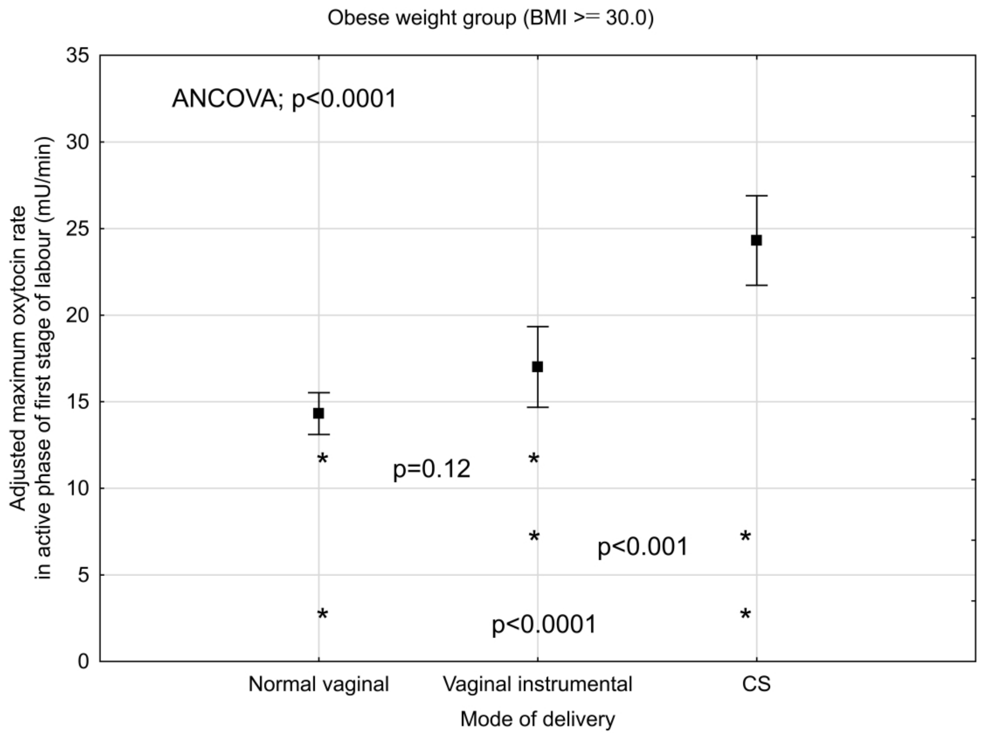

Figure 4. Maximum oxytocin rate in active phase of first stage of labour and mode of delivery in obese, nulliparous women with singleton, term pregnancy with cephalic presentation and augmentation of labour. Plots indicate mean and bars indicate 95% confidence interval. Adjusted for cervical dilatation at start of oxytocin infusion, fetal birth weight and epidural analgesia.

1587x1190mm (96 x 96 DPI)

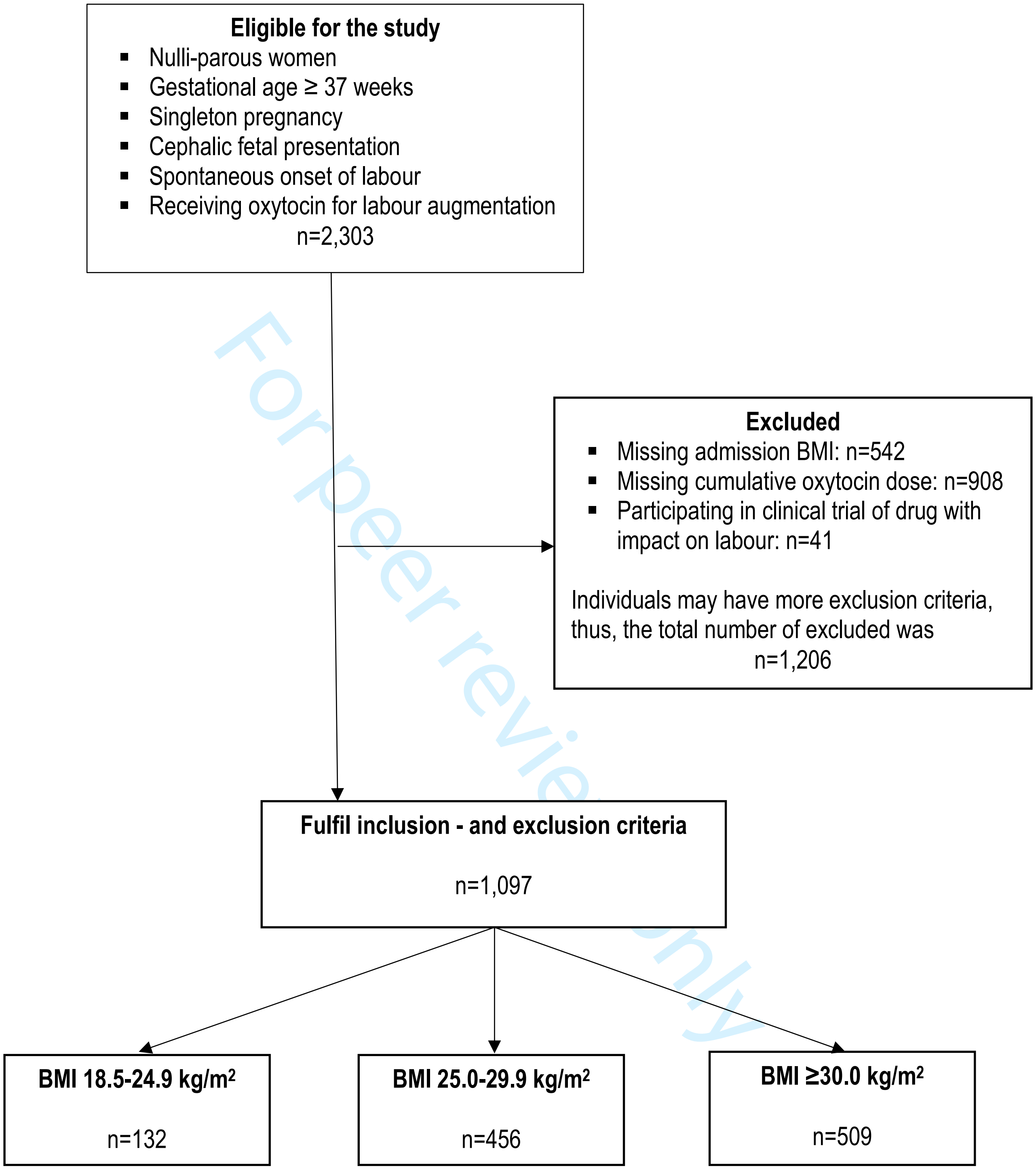

Figure S1. Flow chart of the study population

For peer review only

Table S1. Comparison of maternal, obstetric and neonatal characteristics in nulliparous women with singleton, term pregnancy with cephalic presentation, spontaneous start of labour and treatment with oxytocin infusion for labour augmentation included in the study and those excluded from the study.

	Included n=1,097	Excluded n=1,206	p-value
Maternal characteristics			
Maternal age (years)	28.5 [4.3]	28.5 [4.6]	NS*
Smoking during pregnancy (Yes/No)	35 / 1022 (3.3 / 96.7)	34 / 1091 (3.0 / 97.0)	NS†
Diabetes Mellitus (Yes/No)	12 / 2085 (1.1 / 98.9)	21 / 1185 (1.7 / 98.3)	NS†
Hypertension disorder (including Preeclampsia) (Yes/No)	31/1066 (2.8/97.2)	40 / 1166 (3.3 / 96.7)	NS†
Maternal weight in first trimester (kg)	68.9 [14.1]	68.1 [13.6], n=1,144	NS*
Gestational weight gain (kg)	15.1 [5.8]	14.8 [5.9], n=675	NS*
BMI in first trimester (kg/m^2)	24.8 [4.7]	24.7 [4.6], n=1,139	NS*
BMI at admission in labour (kg/m^2)	30.3 [5.0]	30.3 [5.0], n=664	NS*
Obstetric characteristics			
Gestational age at delivery (days)	282 [7.6]	282 [7.7]	NS*
Mode of delivery:			
Normal vaginal delivery	891 (81.2)	924 (76.6)	<0.01‡
Instrumental vaginal delivery	142 (12.9)	176 (14.6)	
Caesarean section (CS)	64 (5.8)	106 (8.8)	
Obstetric anal sphincter injury (Yes/No)	58 / 1039 (5.3 / 94.7)	49 / 1157 (4.1 / 95.9)	NS†
Indication CS (asphyxia/dystocia)	18 / 46 (28.1 / 71.9)	41 / 65 (38.7 / 61.3)	NS†
Epidural analgesia (Yes/No)	866 / 231 (78.9 / 21.1)	918 / 288 (76.1 / 23.9)	NS†
Postpartum haemorrhage (mL)	470 [368]	513 [406]	<0.001#
Postpartum haemorrhage >1000mL (Yes/No)	81 / 1008 (7.4 / 92.6)	98 / 866 (10.2 / 89.8)	0.03†
Neonatal characteristics			
Fetal birth weight (gram)	3560 [449]	3545 [466]	NS*
Apgar<7 at 5 minutes (Yes/No)	19 / 1078 (1.7 / 98.3)	32 / 1174 /2.7 / 97.3)	NS†
Apgar <4 at 5 minutes (Yes/No)	1 / 1096 (0.1 / 99.9)	4 / 1202 (0.3 / 99.7)	NS†
Umbilical arterial pH <7.0 (Yes/No)	14 / 1083 (1.3 / 98.7)	17 / 1189 (1.4 / 98.6)	NS†

Figures denote mean and [one standard deviation] or number and (%).
*One-way ANOVA; #Mann-Whitney U test; † Pearson's Chi-squared test (df=1). ‡ Pearson's Chi-squared test (df=2).
Level of significance $p<0.05$, NS= Not Significant

STROBE 2007 (v4) checklist of items to be included in reports of observational studies in epidemiology*
Checklist for cohort, case-control, and cross-sectional studies (combined)

Section/Topic	Item #	Recommendation	Reported on page #
**Title and abstract**	1	(*a*) Indicate the study's design with a commonly used term in the title or the abstract	1,2
		(*b*) Provide in the abstract an informative and balanced summary of what was done and what was found	2
**Introduction**			
Background/rationale	2	Explain the scientific background and rationale for the investigation being reported	4
Objectives	3	State specific objectives, including any pre-specified hypotheses	4
**Methods**			
Study design	4	Present key elements of study design early in the paper	5,6
Setting	5	Describe the setting, locations, and relevant dates, including periods of recruitment, exposure, follow-up, and data collection	5,6
Participants	6	(*a*) *Cohort study*—Give the eligibility criteria, and the sources and methods of selection of participants. Describe methods of follow-up<br>*Case-control study*—Give the eligibility criteria, and the sources and methods of case ascertainment and control selection. Give the rationale for the choice of cases and controls<br>*Cross-sectional study*—Give the eligibility criteria, and the sources and methods of selection of participants	5
		(*b*) *Cohort study*—For matched studies, give matching criteria and number of exposed and unexposed<br>*Case-control study*—For matched studies, give matching criteria and the number of controls per case	
Variables	7	Clearly define all outcomes, exposures, predictors, potential confounders, and effect modifiers. Give diagnostic criteria, if applicable	6,7
Data sources/ measurement	8*	For each variable of interest, give sources of data and details of methods of assessment (measurement). Describe comparability of assessment methods if there is more than one group	*6,7*
Bias	9	Describe any efforts to address potential sources of bias	5,6,7
Study size	10	Explain how the study size was arrived at	7
Quantitative variables	11	Explain how quantitative variables were handled in the analyses. If applicable, describe which groupings were chosen and why	6,7
Statistical methods	12	(*a*) Describe all statistical methods, including those used to control for confounding	7
		(*b*) Describe any methods used to examine subgroups and interactions	7
		(*c*) Explain how missing data were addressed	Table 1
		(*d*) *Cohort study*—If applicable, explain how loss to follow-up was addressed<br>*Case-control study*—If applicable, explain how matching of cases and controls was addressed	

For peer review only

		Cross-sectional study—If applicable, describe analytical methods taking account of sampling strategy	
		(*e*) Describe any sensitivity analyses	
**Results**			
Participants	13*	(a) Report numbers of individuals at each stage of study—eg numbers potentially eligible, examined for eligibility, confirmed eligible, included in the study, completing follow-up, and analysed	8, table 1
		(b) Give reasons for non-participation at each stage	5, Suppl Figure S1
		(c) Consider use of a flow diagram	Suppl Figure S1
Descriptive data	14*	(a) Give characteristics of study participants (eg demographic, clinical, social) and information on exposures and potential confounders	8,9 Table 1
		(b) Indicate number of participants with missing data for each variable of interest	Table 1
		(c) *Cohort study*—Summarise follow-up time (eg, average and total amount)	
Outcome data	15*	*Cohort study*—Report numbers of outcome events or summary measures over time	8,9 Table 1
		Case-control study—Report numbers in each exposure category, or summary measures of exposure	
		Cross-sectional study—Report numbers of outcome events or summary measures	
Main results	16	(*a*) Give unadjusted estimates and, if applicable, confounder-adjusted estimates and their precision (eg, 95% confidence interval). Make clear which confounders were adjusted for and why they were included	8,9
		(*b*) Report category boundaries when continuous variables were categorized	6
		(*c*) If relevant, consider translating estimates of relative risk into absolute risk for a meaningful time period	
Other analyses	17	Report other analyses done—eg analyses of subgroups and interactions, and sensitivity analyses	9
**Discussion**			
Key results	18	Summarise key results with reference to study objectives	10,13
Limitations	19	Discuss limitations of the study, taking into account sources of potential bias or imprecision. Discuss both direction and magnitude of any potential bias	10,11,12
Interpretation	20	Give a cautious overall interpretation of results considering objectives, limitations, multiplicity of analyses, results from similar studies, and other relevant evidence	12,13
Generalisability	21	Discuss the generalisability (external validity) of the study results	11
**Other information**			
Funding	22	Give the source of funding and the role of the funders for the present study and, if applicable, for the original study on which the present article is based	7,14

*Give information separately for cases and controls in case-control studies and, if applicable, for exposed and unexposed groups in cohort and cross-sectional studies.
Note: An Explanation and Elaboration article discusses each checklist item and gives methodological background and published examples of transparent reporting. The STROBE checklist is best used in conjunction with this article (freely available on the Web sites of PLoS Medicine at http://www.plosmedicine.org/, Annals of Internal Medicine at http://www.annals.org/, and Epidemiology at http://www.epidem.com/). Information on the STROBE Initiative is available at www.strobe-statement.org.

For peer review only

BMJ Open

Maternal body mass index and oxytocin in augmentation of labour in nulliparous women: a prospective observational study

Journal:	*BMJ Open*
Manuscript ID	bmjopen-2020-044754.R1
Article Type:	Original research
Date Submitted by the Author:	23-Dec-2020
Complete List of Authors:	Ramö Isgren, Anna; Linköping University, Department of Obstetrics and Gynaecology, Department of Biomedical and Clinical Sciences, University Hospital, Linköping<br>Kjolhede, Preben; Linköping University, Departement of Obstetrics and Gynaecology, Department of Biomedical and Clinical Sciences, University Hospital, Linköping<br>Carlhäll, Sara; Linköping University, Departement of Obstetrics and Gynaecology, Department of Biomedical and Clinical Sciences, University Hospital, Linköping<br>Blomberg, Marie; Linköping University, Department of Obstetrics and Gynaecology, Department of Biomedical and Clinical Sciences, University Hospital, Linköping
**Primary Subject Heading**:	Obstetrics and gynaecology
Secondary Subject Heading:	Reproductive medicine
Keywords:	Maternal medicine < OBSTETRICS, Prenatal diagnosis < OBSTETRICS, Fetal medicine < OBSTETRICS

SCHOLARONE™
Manuscripts

I, the Submitting Author has the right to grant and does grant on behalf of all authors of the Work (as defined in the below author licence), an exclusive licence and/or a non-exclusive licence for contributions from authors who are: i) UK Crown employees; ii) where BMJ has agreed a CC-BY licence shall apply, and/or iii) in accordance with the terms applicable for US Federal Government officers or employees acting as part of their official duties; on a worldwide, perpetual, irrevocable, royalty-free basis to BMJ Publishing Group Ltd ("BMJ") its licensees and where the relevant Journal is co-owned by BMJ to the co-owners of the Journal, to publish the Work in this journal and any other BMJ products and to exploit all rights, as set out in our licence.

The Submitting Author accepts and understands that any supply made under these terms is made by BMJ to the Submitting Author unless you are acting as an employee on behalf of your employer or a postgraduate student of an affiliated institution which is paying any applicable article publishing charge ("APC") for Open Access articles. Where the Submitting Author wishes to make the Work available on an Open Access basis (and intends to pay the relevant APC), the terms of reuse of such Open Access shall be governed by a Creative Commons licence – details of these licences and which Creative Commons licence will apply to this Work are set out in our licence referred to above.

Other than as permitted in any relevant BMJ Author's Self Archiving Policies, I confirm this Work has not been accepted for publication elsewhere, is not being considered for publication elsewhere and does not duplicate material already published. I confirm all authors consent to publication of this Work and authorise the granting of this licence.

For peer review only

Maternal body mass index and oxytocin in augmentation of labour in nulliparous women: a prospective observational study

Anna Ramö Isgren, MD, Preben Kjølhede, MD, PhD, Sara Carlhäll, MD, PhD, Marie Blomberg, MD, PhD.

Affiliation

Department of Obstetrics and Gynaecology in Linköping, Department of Biomedical and Clinical Sciences, Linköping University, Sweden

Corresponding author

Marie Blomberg, Professor

Department of Obstetrics and Gynaecology

Linköping UniversitySE-581 85 Linköping Sweden

Telephone number: +46-13288956

E-mail: marie.blomberg@liu.se

Word count: 3198

For peer review only

ABSTRACT

Objective To evaluate oxytocin use for augmentation of labour in relation to body mass index (BMI) on admission to the labour ward, focusing on cumulative oxytocin dose and maximum rate of oxytocin infusion during the first stage of labour.

Design Prospective observational study.

Setting Seven hospitals in Sweden.

Participants 1,097 nulliparous women with singleton cephalic presentation pregnancy, ≥37 weeks of gestation, spontaneous onset of labour and treatment with oxytocin infusion for labour augmentation. The study population was classified into three BMI subgroups on admission to the labour ward: normal weight (18.5-24.9), overweight (25.0-29.9), obese (≥30.0). The cumulative oxytocin dose was measured from the start of oxytocin infusion until the neonate was born.

Primary outcome Cumulative oxytocin dose.

Secondary outcome Maximum rate of oxytocin infusion during the active phase of first stage of labour.

Results The mean cumulative oxytocin dose increased in the BMI groups (normal weight 2278 mU, overweight 3108 mU and obese 4082 mU ($p<0.0001$)). However, when adjusted for the confounders (cervical dilatation when oxytocin infusion was started, fetal birth weight, epidural analgesia) the significant difference was no longer seen. The maximum oxytocin infusion rate during the first stage of labour differed significantly in the BMI groups when adjusted for the confounding factors individually but not when adjusted for all three factors simultaneously. In addition, the maximum oxytocin infusion rate was significantly higher in women with emergency CS compared with women with vaginal delivery.

Conclusions Women with increasing BMI with augmentation of labour received a higher cumulative oxytocin dose and had a higher maximum oxytocin infusion rate during first stage of labour, however, when adjusted for relevant confounders the difference was no longer seen. In the

For peer review only

For peer review only - http://bmjopen.bmj.com/site/about/guidelines.xhtml

future, the guidelines for augmentation of labour with oxytocin infusion might be reconsidered and include modifications related to BMI.

Strengths and limitations of this study

- This is a large, prospective, multicentre study including seven hospitals in Sweden.
- The study population was restricted to nulliparous women in full term pregnancy with spontaneous onset of labour.
- Detailed background characteristics of the study population included pregnancy, obstetric and neonatal characteristics.
- The national Swedish guidelines for the definition of spontaneous start of labour was used which may limit the generalisability of the study.

For peer review only

INTRODUCTION

Maternal obesity is associated with increased risks for obstetric complications.[1-4] In the first stage of labour, obese women have been reported to have a slower progression with increased risks for dystocia which, in turn, are risk factors for chorioamnionitis, emergency Caesarean section (CS) and major postpartum bleeding.[4-14] With a higher incidence of dystocia women with obesity have an increased need for treatment with oxytocin infusion for augmentation during labour. Previous studies have indicated that women with higher body mass index (BMI) receive larger oxytocin infusion doses and higher rates of oxytocin infusion in augmentation and induction of labour.[15-20] However, there is heterogeneity in the design of these studies and the results are not entirely consistent. Today, the guidelines for oxytocin administration in augmentation of labour differ worldwide and none of the guidelines adjust for maternal weight or BMI.[21-23] More research is needed to establish evidence-based clinical guidelines for the use of oxytocin during labour in relation to BMI.

This study evaluates oxytocin use for augmentation of labour in nulliparous women with spontaneous onset at term in relation to maternal BMI on admission to the labour ward. The primary aim was to determine the cumulative oxytocin dose in relation to the woman´s BMI. Secondary aim was to analyse the association between maternal BMI and maximum rate of oxytocin infusion during the active phase of the first stage of labour.

For peer review only - http://bmjopen.bmj.com/site/about/guidelines.xhtml

METHODS

This is a prospective observational multicentre study conducted between April 2017 and February 2019 in all seven departments of Obstetrics and Gynaecology in the southeast health region of Sweden.

Inclusion criteria were nulliparous women with a singleton cephalic presentation pregnancy, ≥37 weeks of gestation with spontaneous onset of labour (i.e. group one in The Ten Group Classification System[24]), and known admission BMI, who were treated with oxytocin infusion for labour augmentation and the cumulative oxytocin dose was measured.

All participating maternity wards adhered to the same national clinical guidelines concerning the definition of spontaneous onset of labour and for augmentation with oxytocin during active labour.[21 25] Likewise, the wards had identical protocols for oxytocin augmentation. The Swedish recommendation for the definition of active spontaneous onset of labour states that at least two out of three of the following criteria have to be fulfilled: spontaneous rupture of the membrane, regular painful contractions (2-3/10 minutes), and the cervix dilated four centimetres or effaced and dilated more than 1 centimetre. In addition to these criteria, the labour should progress within the following two hours.[25] The recommendation for initiation of augmentation with oxytocin during the active phase of the first stage of labour was defined as delayed progress of three hours from the expected 1 cm/hour cervical dilatation and during the second stage defined as no descent for one hour, or active pushing for more than 30 minutes.[21] The guidelines for starting the oxytocin infusion also entails continuously cardiotocography (CTG), for registration of the fetal heart rate and monitoring contractions of the uterus.

Oxytocin was administered as an intravenous infusion through a peripheral venous catheter by an electronic infusion pump (B BRAUN Infusomat® Space or Alaris™ GP Plus). A standard solution for infusion of oxytocin was prepared by adding 1 mL (containing 5 units of oxytocin) to 500 mL of 0.9% sodium chloride, reaching a concentration of 10 mU/mL. The infusion was

For peer review only - http://bmjopen.bmj.com/site/about/guidelines.xhtml

routinely started at an infusion rate of 3.3 mU/min and the rate was increased every 20 minutes by 3.3 mU/min at a time until 4-5 contractions/10 minutes were achieved and/or normal progress of labour occurred according to the partograph, a graphic record of the progress of labour. Hyperstimulation was defined as >5 contractions/10 minutes.[21] The cumulative oxytocin dose was automatically registered by the infusion pump and was measured from the start of the oxytocin infusion until the neonate was born. The attending midwife registered the rate of oxytocin infusion manually in the partograph continuously during labour, and took the reading from the pump after delivery for registration of the cumulative oxytocin dose.

Maternal height was reported or measured at the first antenatal visit and maternal weight was measured at admission to the labour ward, which enabled calculation of admission BMI (kg/m^2). The study population was classified into three subgroups according to the World Health Organization (WHO) BMI classification based on their BMI on admission to the labour ward: normal weight (BMI 18.5-24.9), overweight (BMI 25.0-29.9), and obese (BMI ≥30.0).

Demographic and clinical data comprising maternal, obstetric and neonatal characteristics were prospectively recorded in standardised medical records (Obstetrix, Cerner). The maternal characteristics consisted of age, smoking habit, diabetes mellitus, hypertension disorder (including preeclampsia), and maternal weight in the first trimester and at delivery. The obstetric characteristics included gestational length at delivery, epidural analgesia, mode of delivery, occurrence of obstetric anal sphincter injury, and postpartum haemorrhage. In addition, the obstetric characteristics included cervical dilatation at the start of oxytocin augmentation subdivided into three categories: < 6 cm, 6-10 cm and fully dilated,[26] the time from the start of active labour until the start of oxytocin augmentation, the time from the start of oxytocin augmentation until delivery, the cumulative oxytocin dose from the start of oxytocin infusion to delivery, and the maximum oxytocin infusion rate during the first stage of active labour.

The neonatal characteristics comprised fetal birth weight, Apgar score (<7 and <4 at 5 minutes) and umbilical arterial pH <7.0.

For peer review only - http://bmjopen.bmj.com/site/about/guidelines.xhtml

Statistics

Sample size estimation was based on the cumulative oxytocin dose, given the assumptions that the minimal clinically important difference in cumulative oxytocin dose, between groups was 1200 mU, and that the standard deviation of the cumulative oxytocin dose was 3000 mU. Thus, in order to detect a statistically significant difference at a 5% level with 80% power the sample size was estimated to be approximately 100 in each group.

Data are presented as mean and one standard deviation (SD), or number and per cent. Comparisons of nominal data were conducted by means of Pearson Chi-squared tests. Continuous data were compared using one-way analysis of variance (ANOVA) tests for normally distributed data and Kruskal-Wallis ANOVA and Mann-Whitney U-tests for non-normally distributed data in the univariate analyses. The outcome measures were evaluated in multivariate analyses by means of two-way ANOVA or analysis of covariance (ANCOVA), adjusting for the confounding factors cervical dilation at start of oxytocin infusion, categorised in < 6 cm, 6-10 cm, and fully dilated, fetal birth weight (as a continuous variable) and use of epidural analgesia (dichotomised in yes or no). In the multivariate analyses, the non-normally distributed continuous data variables underwent logarithmic transformation. Post-hoc tests were performed by means of Tukey's honestly significant difference (HSD) test. The level of significance was set at $p<0.05$ for two-tailed tests. Statistical analyses were performed using the software TIBCO Statistica™, version 13.5 (TIBCO Software Inc, Palo Alto, CA 94303, USA).

Patient and public involvement

Patients or the public were not involved in the design, or conduct, or reporting or dissemination plans of our research.

For peer review only

For peer review only - http://bmjopen.bmj.com/site/about/guidelines.xhtml

RESULTS

The selection of the study population is presented in the flow chart (online Supplementary Figure S1). A total of 1,097 women were included in the study. Maternal, obstetric and neonatal characteristics of the study population in relation to BMI group are presented in Table 1.

For peer review only

For peer review only - http://bmjopen.bmj.com/site/about/guidelines.xhtml

Table 1. Maternal, obstetric and neonatal characteristics in nulliparous women with singleton, term pregnancy with cephalic presentation and augmentation of labour with oxytocin infusion according to admission in labour body mass index.

		BMI 18.5-24.9 (n= 132)	BMI 25.0-29.9 (n=456)	BMI ≥30.0 (n=509)	p-values
Maternal characteristics					
Maternal age (years)		28.1 [4.1]	28.8 [4.4]	28.2 [4.2]	0.09*
Smoking during pregnancy		5 (3.8)	11 (2.4)	19 (3.7)	0.46#
Diabetes mellitus		0 (0)	4 (0.9)	8 (1.6)	0.25#
Hypertension disorder (including preeclampsia)		0 (0)	7 (1.5)	24 (4.7)	<0.01#
Maternal weight in first trimester (kg)		56.1 [6.1]	62.4 [6.7]	78.3 [14.4]	<0.0001*
Gestational weight gain (kg)		11.1 [3.6]	14.2 [4.4]	16.9 [6.6]	<0.0001*
Obstetric characteristics					
Gestational age at delivery (days)		281.4 [7.8]	281.5 [7.7]	282.4 [7.5]	0.13*
Time from active labour until start of oxytocin (h)		8.6 [4.0] (n=105)	9.0 [5.3] (n=385)	8.4 [4.6] (n=422)	0.23†
Time from start of oxytocin until delivery (h)		3.0 [2.7]	3.8 [3.4]	5.0 [4.1]	<0.0001†
Cervical dilatation at start of oxytocin	< 6 cm	16 (12.1)	87 (19.1)	144 (28.3)	<0.0001¤
	6-10 cm	34 (25.8)	142 (31.1)	207 (40.7)	
	Fully dilated	82 (62.1)	227 (49.8)	158 (31.0)	
Mode of delivery:	Normal vaginal delivery	107 (81.1)	379 (83.1)	405 (79.6)	<0.01¤
	Instrumental vaginal delivery	22 (16.7)	59 (12.9)	61 (12.0)	
	Caesarean section	3 (2.3)	18 (3.9)	43 (8.4)	
Obstetric anal sphincter injury		8 (6.1)	22 (4.8)	28 (5.5)	0.82#
Postpartum haemorrhage (mL)		456 [350]	454 [378]	488 [363]	<0.01†
Postpartum haemorrhage > 1000 mL		10 (7.6)	31 (6.9)	40 (7.9)	0.83#
Epidural analgesia (no. of women)		96 (72.7)	355 (77.9)	415 (81.5)	0.07#
Neonatal characteristics					
Fetal birth weight (gram)		3329 [351]	3515 [420]	3661 [467]	<0.0001*
Apgar score <7 at 5 minutes (no. of infants)		1 (0.8)	7 (1.5)	11 (2.2)	0.50#
Apgar score <4 at 5 minutes (no. of infants)		0 (0)	0 (0)	1 (0.2)	0.56#
Umbilical arterial pH <7.0 (no. of infants)		0 (0) (n=110)	7 (1.5) (n=393)	7 (1.4) (n=442)	0.37#

Figures denote mean and [one standard deviation] or number and (%)

* One-way ANOVA; † Kruskal-Wallis ANOVA; # Pearson Chi-square (df=2); ¤ Pearson Chi-square (df= 4)

For peer review only

For peer review only - http://bmjopen.bmj.com/site/about/guidelines.xhtml

As shown in Table 2, the cumulative oxytocin dose increased significantly with increasing BMI group (normal weight, mean 2278 mU [2748 mU]; overweight, mean 3108 mU [3839 mU], and obese, mean 4082 mU [4895 mU]; Kruskal-Wallis ANOVA p<0.0001). However, when adjusting for all three confounders, the significant difference was no longer seen between the three BMI groups (Table 2).

For peer review only

For peer review only - http://bmjopen.bmj.com/site/about/guidelines.xhtml

Table 2. Cumulative oxytocin dose and maximum rate of oxytocin during active phase of first stage of labour according to body mass index.

				ANOVA / ANCOVA between BMI groups, p-value				
	BMI 18.5-24.9	BMI 25.0-29.5	BMI ≥30	Crude*	Adjusted [1]	Adjusted [2]	Adjusted [3]	Adjusted [4]
	(n=132)	(n=456)	(n=509)					
Cumulative oxytocin dose (mU)	2278 [2748]	3108 [3839]	4082 [4895]	<0.0001	0.42	<0.001	<0.0001	0.88
Independent predictive factors:								
Cervical dilatation					<0.0001			<0.0001
Fetal birth weight						<0.0001		<0.0001
Epidural analgesia							<0.0001	<0.0001
	(n=49)	(n=220)	(n=347)					
Maximum rate of oxytocin during active phase of first stage of labour (mU/min)	12.7 [7.1]	13.6 [9.1]	15.5 [9.5]	<0.01	<0.05	<0.05	0.01	0.10
Independent predictive factor:								
Cervical dilatation					<0.0001			<0.0001
Fetal birth weight						<0.01		<0.0001
Epidural analgesia							<0.01	<0.01

Figures denote mean and [SD]

* Kruskal-Wallis ANOVA

1 Adjusted for cervical dilatation at start of oxytocin infusion

2 Adjusted for fetal birth weight

3 Adjusted for epidural analgesia

4 Adjusted simultaneously for 1, 2, and 3.

For peer review only

For peer review only - http://bmjopen.bmj.com/site/about/guidelines.xhtml

Each of the three confounders were strong independent predictors of the cumulative oxytocin dose, but adjusted simultaneously together they equalise the differences in cumulative oxytocin dose completely between the BMI groups (Figure 1).

The maximum rate of oxytocin infusion during the active phase of the first stage of labour differed significantly in the BMI groups in the univariate analyses (normal weight (n=49), mean 12.7 mU/min [7.1 mU/min], overweight (n=220), mean 13.6 mU/min [9.1 mU/min], and obese (n=347), mean 15.5 mU/min [9.5 mU/min]; Kruskal-Wallis ANOVA, $p<0.01$). Individually, the confounding factors did not influence this, but when the analysis was conducted with all three confounding factors simultaneously, the significant difference between the BMI groups was no longer seen (Table 2, Figure 2).

When analysing the subgroup of women with overweight and obesity and known maximum rate of oxytocin infusion during the active phase of the first stage of labour, the maximum infusion rate was significantly associated with mode of delivery in both BMI groups when adjusted simultaneously for cervical dilation, fetal birth weight and epidural analgesia (Figures 3, 4).

The time from active labour until the start of oxytocin infusion did not differ between the BMI groups (Kruskal-Wallis ANOVA, $p=0.23$), (Table 1), even when adjusted for fetal birth weight and epidural analgesia (ANCOVA, $p=0.18$). A significantly higher proportion of women with obesity ended with an emergency CS compared with normal weight and overweight women (8.4% versus 2.3% and 3.9%, respectively) (Pearson Chi-squared test; $df=2$, $p<0.01$) whereas no significant differences were seen between the BMI groups in women with a normal vaginal delivery or instrumental vaginal delivery.

The analysis of the women included in the study group versus those who were excluded mainly because of missing cumulative oxytocin dose showed similar proportions of characteristics, including indication for CS, in the two groups except for the rate of CS (5.8% in the study group versus 8.8% in the group of excluded women) and the occurrence of postpartum haemorrhage exceeding 1000 mL (7.4% versus 10.2%) (Online Supplementary Table S1). Further analysis was

For peer review only

For peer review only - http://bmjopen.bmj.com/site/about/guidelines.xhtml

conducted between the women who had CS with registration of cumulative oxytocin dose (n=64) and those women who had CS but oxytocin dose was not registered (n=106). The degree of cervical dilation when oxytocin augmentation started did not differ nor did the time from the start of active labour to the start of infusion of oxytocin differ (data not shown). The time from the start of oxytocin augmentation to delivery was significantly shorter in those women where the cumulative oxytocin dose was registered (mean 4.1 h (3.6 h) versus 8.6 h (5.1 h) (Mann-Whitney U-test, $p<0.01$)).

For peer review only

For peer review only - http://bmjopen.bmj.com/site/about/guidelines.xhtml

DISCUSSION

Principal findings

The present study showed that nulliparous women at term with spontaneous onset and augmentation of labour in unadjusted analysis received a higher cumulative oxytocin dose and had a higher maximum oxytocin infusion rate at first stage of labour with increasing BMI on admission to the labour ward. After adjusting for cervical dilation at start of oxytocin infusion, fetal birth weight and epidural analgesia, the significant difference was no longer seen. In addition, the maximum oxytocin infusion rate in first stage of labour was significantly higher in women with emergency CS compared with vaginal delivery.

Strengths and limitations

The major strength of the study is the large number of participants restricted to nulliparous women with spontaneous onset of labour. The multicentre design made it possible to include women with different sociodemographic backgrounds, including both urban and rural areas. In addition, the labour wards included in the study had the same clinical guidelines for obstetric care.

The study has some limitations. Since there are no known cut-off values for BMI during pregnancy we used the definition according to the WHO criteria. There are some limitations with the use of admission in labour BMI. Except increased fat mass, increased volume of extravascular fluid and blood, increased weight from maternal tissue from breast and uterus, admission BMI also includes the weight of the fetus, placenta and amniotic fluid. However, these limitations do not affect the purpose of this study, which was to find a relationship between BMI at admission to the labour ward, and the cumulative oxytocin dose and maximum oxytocin infusion rate in the first stage of labour.

There might be a risk of selection bias since the cumulative oxytocin dose was not registered for all women. However, in order to determine the potential scope of selection bias we compared

For peer review only

the study group with the women that were excluded from the study. The data showed that the groups were similar except for the proportions of CS and postpartum haemorrhage, which were much higher in the excluded group. A plausible explanation for this discrepancy might be the time pressure that the staff were exposed to when a delivery was converted to an emergency CS or heavy postpartum bleeding occurred, and thus they forgot to record the cumulative oxytocin dose. All these aspects speak against selection bias.

Another limitation is our definition of the start of active labour. We used the national Swedish guidelines for the definition of spontaneous start of labour. There is no consensus worldwide either of the definition of spontaneous start of active labour or the definition of dystocia and this may limit the generalizability of the study. All participating sites in this study have well established routines if hyperstimulation occurs (e.g. reduce or stop the oxytocin infusion); unfortunately, we did not have the exact number of periods of hyperstimulation registered as the CTG-traces were not scrutinised in the study.

Interpretation in relation to other studies

This prospective study showed that women with increasing BMI received a higher cumulative dose of oxytocin compared with normal weight women, however, when adjusting for cervical dilation at start of oxytocin infusion no significant difference between the BMI groups were seen. Soni et al also studied women with spontaneous start of labour and treatment with oxytocin for arrest of dilation. They found that the total amount of oxytocin administrated was significantly higher in a group of women with admission BMI > 35 (n=23) compared with women in three lower BMI groups. The BMI-groups did not differ according to EDA or birth weight, however, the cervical dilatation at arrest differed significantly and were not adjusted for in the study.[15]

The present study showed that women with increasing BMI received a higher maximum infusion rate of oxytocin for augmentation in the first stage of labour even after adjustment for cervical dilation at start of oxytocin infusion. When additionally adjusting for fetal birth weight and epidural

For peer review only - http://bmjopen.bmj.com/site/about/guidelines.xhtml

analgesia the difference was no longer seen. The maximum oxytocin infusion rate in the first stage of labour in relation to maternal BMI has not been studied before in women with spontaneous start of labour. However, Hill et al. showed in induced labour that obese women (BMI >40) had a significantly higher mean maximum oxytocin infusion rate during first stage of labour compared with a group of lean women (BMI<28), matched for gestational age, fetal birth weight and cervical dilatation and fetal station at admission.[16] Adam´s et al studied oxytocin use in women with induced and spontaneous onset of labour and demonstrated that women with class III obesity had an increased risk for an oxytocin rate >20 mU/min, from admission until vaginal delivery, compared with lower BMI groups. However, in a sub analysis on spontaneous onset of labour, no significance was seen.[19]

The present study showed that women with obesity started oxytocin infusion at an earlier cervical stage compared with lower BMI groups. The reason for this can be discussed. Earlier studies have showed a longer first, but not second, stage of labour in obese women.[4 8 14] The time point when the oxytocin infusion starts equals when labour dystocia is diagnosed. Obese women received oxytocin infusion at an earlier cervical dilatation stage which could indicate an abnormal uterine contractility. Underlying pathophysiological mechanisms and ineffective uterine contractility among obese women have been studied. From biopsies, Zhang et al showed that the myometrium from obese women undergoing elective CS at term contracted with less frequency and force compared with the myometrium from normal weight women.[6] Other studies have indicated a different metabolic profile in obese women that could affect the contraction strength in the myometrium in an inhibitory way.[27 28]

Clinical implications

There is evidence that oxytocin administration increases the risk for uterine hyperstimulation during labour, which can lead to fetal heart rate abnormalities. Furthermore, observational studies have shown an association between oxytocin use and increased risk for postpartum haemorrhage.[29]

For peer review only - http://bmjopen.bmj.com/site/about/guidelines.xhtml

Therefore, clinicians must use oxytocin with caution with respect to the potential risks and simultaneously be aware of the consequence of a CS due to dystocia and insufficient treatment with oxytocin in obese women. Our study showed that the maximum oxytocin infusion rate was significantly increased in overweight and obese women with emergency CS compared with vaginal deliveries. The reason for this is unclear but the explanation could be labour dystocia that did not respond to oxytocin treatment, or fetal distress due to uterine hyperstimulation. Further research is needed to explore why women respond differently to oxytocin treatment. Perhaps there is a different bodily distribution of administrated oxytocin in different BMI groups.

Conclusion and future directions

This large prospective study showed that women with increasing BMI received a higher cumulative oxytocin dose and higher maximum oxytocin infusion rate during the active phase of first stage of labour, however, when adjusting for cervical dilation at start of oxytocin infusion, fetal birth weight and use of epidural analgesia, the significant difference disappeared.

Oxytocin is one of the most commonly used drugs in the maternity wards and obesity is an increasing and major concern in obstetric healthcare. There are still knowledge gaps and further research is needed regarding the pathophysiological mechanisms in obese women affecting the dosage and duration of oxytocin in labour augmentation. In order to optimise treatment of labour dystocia, the patient care has to be individualized and the guidelines for augmentation of labour with oxytocin infusion might in the future be reconsidered and include modifications related to maternal BMI.

For peer review only - http://bmjopen.bmj.com/site/about/guidelines.xhtml

Acknowledgements

We would like to thank the midwives and staff of the participating hospitals for collecting data.

Contribution to Authorship

All authors (ARI, PK, SC, MB) conceived the study idea, interpreted the data analysis and critically revised the manuscript. ARI acquired the data and PK and ARI performed the data analysis. All the authors (ARI, PK, SC, MB) agree to take responsibility for the work.

Funding

This work was supported by the Medical Research Council of Southeast Sweden, FORSS (grant no. FORSS-756621).Unrestricted grants were obtained from the County Council of Östergötland and Linköping University, Sweden. The funders were not involved in the development of the study from idea to project plan, implementation, analysis of data or writing of the manuscript.

Competing interests

The authors have no conflicts of interest to disclose.

Patient consent for publication

Not required

Details of Ethical Approval

The Regional Ethical Review Board in Linköping, Sweden approved the study (Dnr 2017/277-31), need for informed consent has been waived for the study from ethics committee. All methods were performed in accordance with the relevant guidelines and regulations.

Data availability statement

Data are available upon reasonable request and according to Swedish legislation. Data statement section, technical appendix, statistical code and dataset available from the authors.

For peer review only

REFERENCES

1. Cedergren MI. Maternal morbid obesity and the risk of adverse pregnancy outcome. *Obstet Gynecol* 2004;103(2):219-24. doi: 10.1097/01.AOG.0000107291.46159.00
2. Baeten JM, Bukusi EA, Lambe M. Pregnancy complications and outcomes among overweight and obese nulliparous women. *American journal of public health* 2001;91(3):436-40. doi: 10.2105/ajph.91.3.436 [published Online First: 2001/03/10]
3. Blomberg M. Maternal obesity and risk of postpartum hemorrhage. *Obstet Gynecol* 2011;118(3):561-8. doi: 10.1097/AOG.0b013e31822a6c59 [published Online First: 2011/08/24]
4. Carlhall S, Kallen K, Blomberg M. Maternal body mass index and duration of labor. *European journal of obstetrics, gynecology, and reproductive biology* 2013;171(1):49-53. doi: 10.1016/j.ejogrb.2013.08.021 [published Online First: 2013/09/18]
5. Hilliard AM, Chauhan SP, Zhao Y, et al. Effect of obesity on length of labor in nulliparous women. *American journal of perinatology* 2012;29(2):127-32. doi: 10.1055/s-0031-1295653 [published Online First: 2011/11/23]
6. Zhang J, Bricker L, Wray S, et al. Poor uterine contractility in obese women. *BJOG : an international journal of obstetrics and gynaecology* 2007;114(3):343-8. doi: 10.1111/j.1471-0528.2006.01233.x [published Online First: 2007/01/31]
7. Kominiarek MA, Zhang J, Vanveldhuisen P, et al. Contemporary labor patterns: the impact of maternal body mass index. *Am J Obstet Gynecol* 2011;205(3):244 e1-8. doi: 10.1016/j.ajog.2011.06.014
8. Vahratian A, Zhang J, Troendle JF, et al. Maternal prepregnancy overweight and obesity and the pattern of labor progression in term nulliparous women. *Obstet Gynecol* 2004;104(5 Pt 1):943-51. doi: 10.1097/01.aog.0000142713.53197.91 [published Online First: 2004/11/02]
9. Norman SM, Tuuli MG, Odibo AO, et al. The effects of obesity on the first stage of labor. *Obstet Gynecol* 2012;120(1):130-5. doi: 10.1097/AOG.0b013e318259589c [published Online First: 2012/08/24]
10. Verdiales M, Pacheco C, Cohen WR. The effect of maternal obesity on the course of labor. *Journal of perinatal medicine* 2009;37(6):651-5. doi: 10.1515/jpm.2009.110 [published Online First: 2009/07/14]
11. Cedergren MI. Non-elective caesarean delivery due to ineffective uterine contractility or due to obstructed labour in relation to maternal body mass index. *European journal of obstetrics, gynecology, and reproductive biology* 2009;145(2):163-6. doi: 10.1016/j.ejogrb.2009.05.022 [published Online First: 2009/06/16]
12. Cheng YW, Shaffer BL, Bryant AS, et al. Length of the first stage of labor and associated perinatal outcomes in nulliparous women. *Obstet Gynecol* 2010;116(5):1127-35. doi: 10.1097/AOG.0b013e3181f5eaf0 [published Online First: 2010/10/23]
13. Nyflot LT, Stray-Pedersen B, Forsen L, et al. Duration of labor and the risk of severe postpartum hemorrhage: A case-control study. *PloS one* 2017;12(4):e0175306. doi: 10.1371/journal.pone.0175306 [published Online First: 2017/04/07]
14. Rodriguez-Mesa N, Robles-Benayas P, Rodriguez-Lopez Y, et al. Influence of Body Mass Index on Gestation and Delivery in Nulliparous Women: A Cohort Study. *Int J Environ Res Public Health* 2019;16(11) doi: 10.3390/ijerph16112015 [published Online First: 2019/06/09]
15. Soni S, Chivan N, Cohen WR. Effect of maternal body mass index on oxytocin treatment for arrest of dilatation. *Journal of perinatal medicine* 2013;41(5):517-21. doi: 10.1515/jpm-2013-0024 [published Online First: 2013/03/22]
16. Hill M, Reed KL, Cohen WR. Oxytocin utilization for labor induction in obese and lean women. *Journal of perinatal medicine* 2015;43(6):703-6. doi: 10.1515/jpm-2014-0134 [published Online First: 2014/09/16]

For peer review only - http://bmjopen.bmj.com/site/about/guidelines.xhtml

17. Pevzner L, Powers BL, Rayburn WF, et al. Effects of maternal obesity on duration and outcomes of prostaglandin cervical ripening and labor induction. *Obstet Gynecol* 2009;114(6):1315-21. doi: 10.1097/AOG.0b013e3181bfb39f [published Online First: 2009/11/26]
18. Roloff K, Peng S, Sanchez-Ramos L, et al. Cumulative oxytocin dose during induction of labor according to maternal body mass index. *International journal of gynaecology and obstetrics: the official organ of the International Federation of Gynaecology and Obstetrics* 2015;131(1):54-8. doi: 10.1016/j.ijgo.2015.04.038 [published Online First: 2015/07/27]
19. Adams AD, Coviello EM, Drassinower D. The Effect of Maternal Obesity on Oxytocin Requirements to Achieve Vaginal Delivery. *American journal of perinatology* 2020;37(4):349-56. doi: 10.1055/s-0039-1694982 [published Online First: 2019/08/21]
20. Subramaniam A, Owen J, Campbell SB, et al. Maternal body mass index and oxytocin exposure in nulliparous women: is there an interaction associated with maternal and neonatal morbidities? *J Matern Fetal Neonatal Med* 2018;31(15):2013-18. doi: 10.1080/14767058.2017.1334046 [published Online First: 2017/05/24]
21. Indication for augmentation with oxytocin during active labour. National Board of Health and Welfare. In Swedish: Indikation för värkstimulering med oxytocin under aktiv förlossning. Rapport från samarbetsprojektet Nationella Medicinska Indikationer; 2011. https://www.socialstyrelsen.se/./nationella-indikationer-varkstimulering-oxytocin.pdf. [Accessed January 2017]
22. Page K, McCool WF, Guidera M. Examination of the Pharmacology of Oxytocin and Clinical Guidelines for Use in Labor. *Journal of midwifery & women's health* 2017;62(4):425-33. doi: 10.1111/jmwh.12610 [published Online First: 2017/07/14]
23. WHO recommendations for augmentation of labour.Geneva:World Health Organization 2014. https://apps.who.int/iris/bitstream/handle/10665/112825/9789241507363_eng.pdf?sequence=1[Accessed January 2017]
24. Robson M. The Ten Group Classification System (TGCS) - a common starting point for more detailed analysis. *BJOG : an international journal of obstetrics and gynaecology* 2015;122(5):701. doi: 10.1111/1471-0528.13267 [published Online First: 2015/01/21]
25. Wiklund I, Ahlberg M, Pettersson K, et al. Definition av etablerat förlossningsarbete (Swedish title).SFOG.Svenska barnmorskeförbundet 2015. https://storage.googleapis.com/barnmorskeforbundet-se/uploads/2015/03/Definition-etablerat-forlossningsarbete-2015.pdf [Accessed January 2017]
26. Zhang J, Landy HJ, Branch DW, et al. Contemporary patterns of spontaneous labor with normal neonatal outcomes. *Obstet Gynecol* 2010;116(6):1281-7. doi: 10.1097/AOG.0b013e3181fdef6e
27. Hehir MP, Morrison JJ. The adipokine apelin and human uterine contractility. *American journal of obstetrics and gynecology* 2012;206(4):359.e1-5. doi: 10.1016/j.ajog.2012.01.032 [published Online First: 2012/03/01]
28. Moynihan AT, Hehir MP, Glavey SV, et al. Inhibitory effect of leptin on human uterine contractility in vitro. *American journal of obstetrics and gynecology* 2006;195(2):504-9. doi: 10.1016/j.ajog.2006.01.106 [published Online First: 2006/05/02]
29. Rousseau A, Burguet A. Oxytocin administration during spontaneous labor: Guidelines for clinical practice. Chapter 5: Maternal risk and adverse effects of using oxytocin augmentation during spontaneous labor. *J Gynecol Obstet Hum Reprod* 2017;46(6):509-21. doi: 10.1016/j.jogoh.2017.04.009 [published Online First: 2017/05/06]

For peer review only - http://bmjopen.bmj.com/site/about/guidelines.xhtml

Figure legends

Figure 1.Cumulative oxytocin dose in nulliparous women with singleton, term, pregnancy with cephalic presentation and augmentation of labour according to admission body mass index.

Plots indicate mean and bars indicate 95% confidence interval. Adjusted for cervical dilatation at start of oxytocin infusion, fetal birth weight and epidural analgesia.

Figure 2. Maximum oxytocin infusion rate in first stage of labour in nulliparous women with singleton, term, pregnancy with cephalic presentation and augmentation of labour according to admission body mass index.

Plots indicate mean and bars indicate 95% confidence interval. Adjusted for cervical dilatation at start of oxytocin infusion, fetal birth weight and epidural analgesia.

Figure 3. Maximum oxytocin rate in active phase of first stage of labour and mode of delivery in overweight, nulliparous women with singleton, term pregnancy with cephalic presentation and augmentation of labour. Plots indicate mean and bars indicate 95% confidence interval. Adjusted for cervical dilatation at start of oxytocin infusion, fetal birth weight and epidural analgesia.

Figure 4. Maximum oxytocin rate in active phase of first stage of labour and mode of delivery in obese, nulliparous women with singleton, term pregnancy with cephalic presentation and augmentation of labour. Plots indicate mean and bars indicate 95% confidence interval. Adjusted for cervical dilatation at start of oxytocin infusion, fetal birth weight and epidural analgesia.

Legends to Supplementary Files

Supplementary Figure S1. Flow chart of the study population

Supplementary Table S1. Comparison of maternal, obstetric and neonatal characteristics in nulliparous women with singleton, term pregnancy with cephalic presentation, spontaneous start of

For peer review only - http://bmjopen.bmj.com/site/about/guidelines.xhtml

labour and treatment with oxytocin infusion for labour augmentation included in the study and those excluded from the study.

For peer review only

For peer review only - http://bmjopen.bmj.com/site/about/guidelines.xhtml

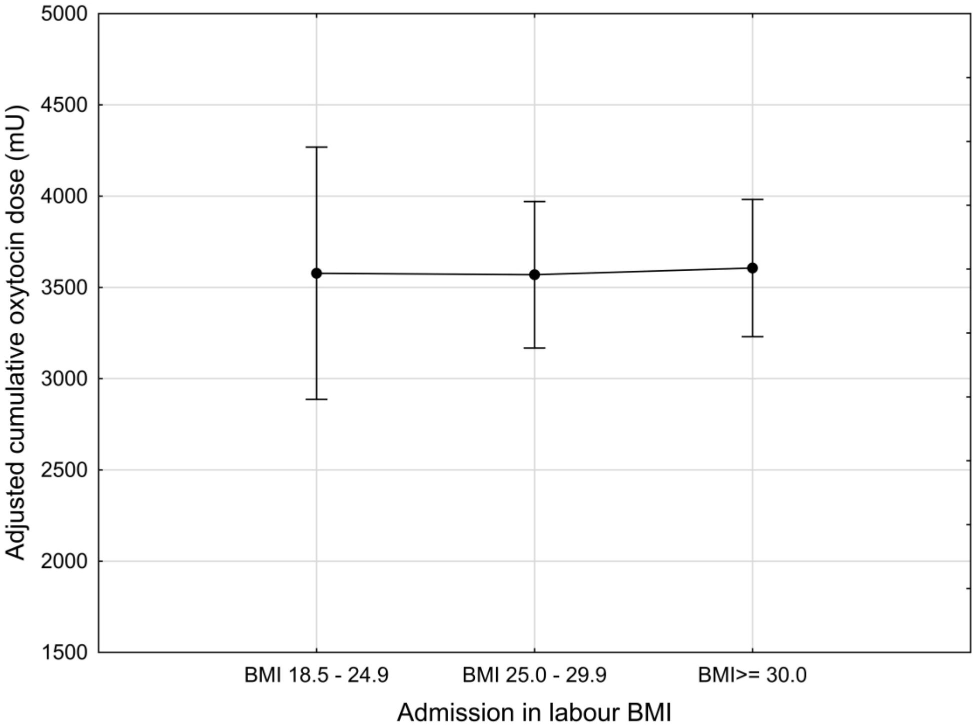

Figure 1.Cumulative oxytocin dose in nulliparous women with singleton, term, pregnancy with cephalic presentation and augmentation of labour according to admission body mass index. Plots indicate mean and bars indicate 95% confidence interval. Adjusted for cervical dilatation at start of oxytocin infusion, fetal birth weight and epidural analgesia.

1587x1190mm (96 x 96 DPI)

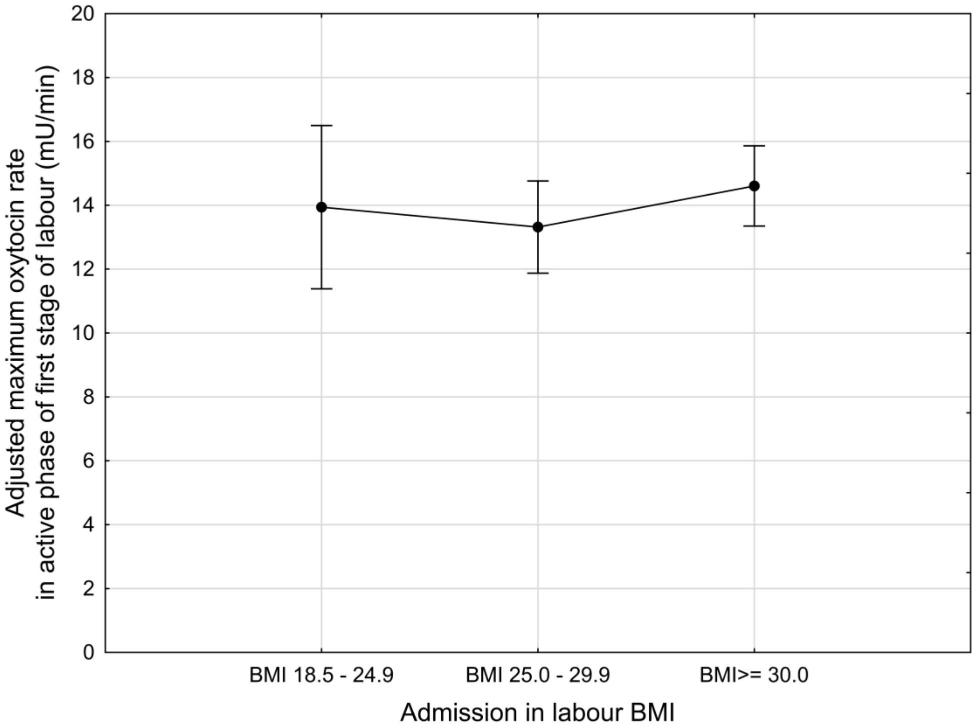

Figure 2. Maximum oxytocin infusion rate in first stage of labour in nulliparous women with singleton, term, pregnancy with cephalic presentation and augmentation of labour according to admission body mass index. Plots indicate mean and bars indicate 95% confidence interval. Adjusted for cervical dilatation at start of oxytocin infusion, fetal birth weight and epidural analgesia.

1587x1190mm (96 x 96 DPI)

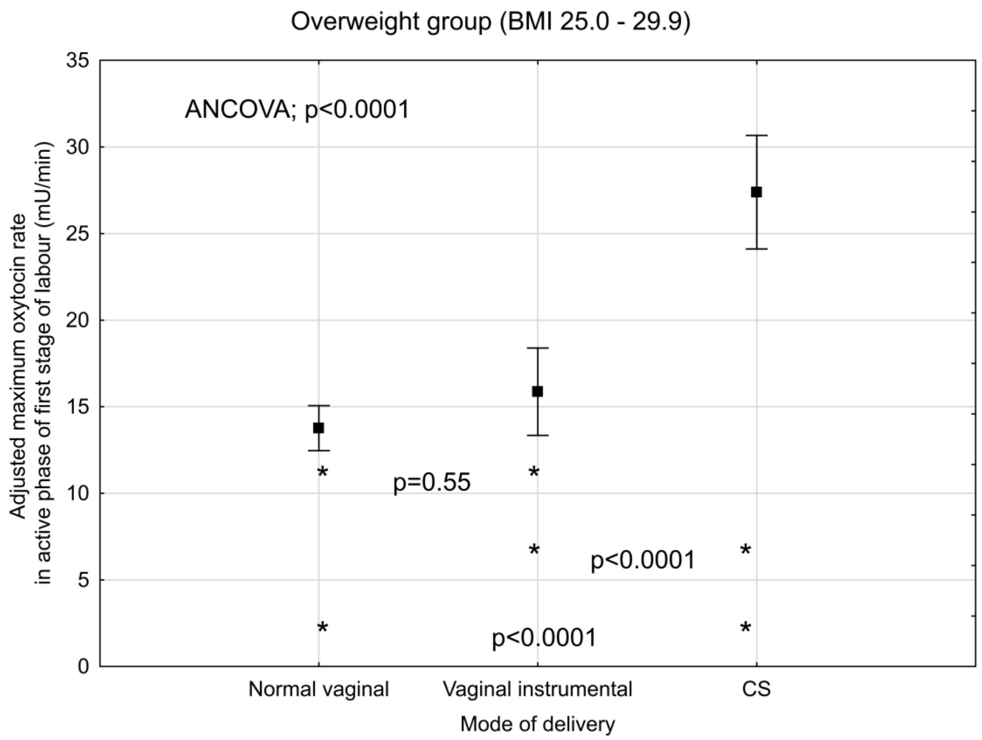

Figure 3. Maximum oxytocin rate in active phase of first stage of labour and mode of delivery in overweight, nulliparous women with singleton, term pregnancy with cephalic presentation and augmentation of labour. Plots indicate mean and bars indicate 95% confidence interval. Adjusted for cervical dilatation at start of oxytocin infusion, fetal birth weight and epidural analgesia

1587x1190mm (96 x 96 DPI)

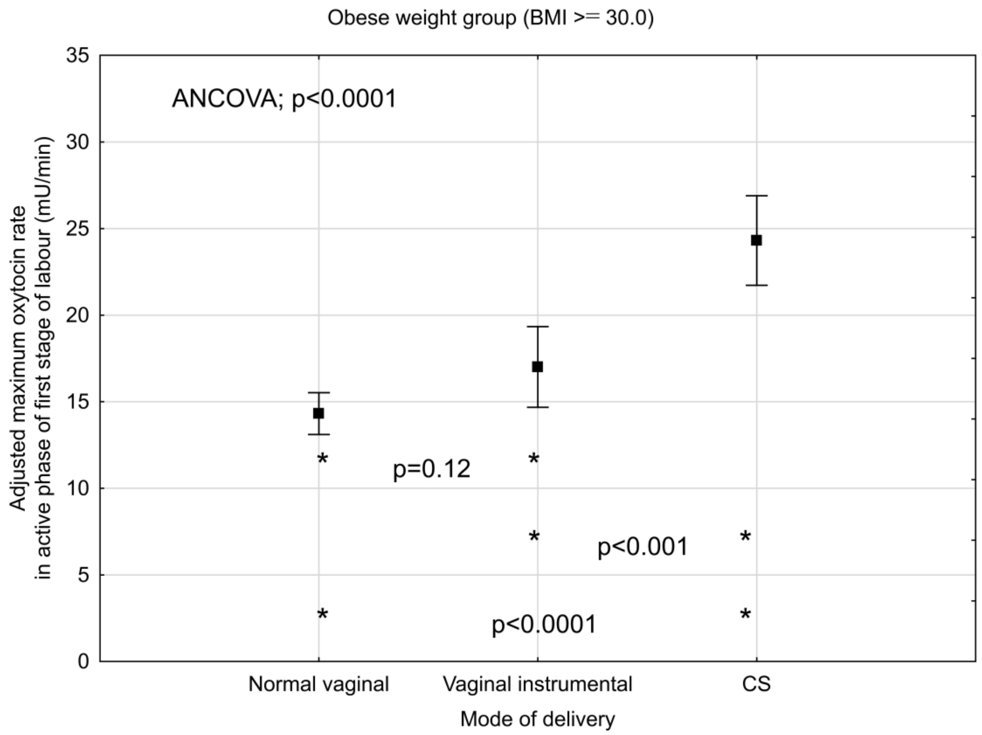

Figure 4. Maximum oxytocin rate in active phase of first stage of labour and mode of delivery in obese, nulliparous women with singleton, term pregnancy with cephalic presentation and augmentation of labour. Plots indicate mean and bars indicate 95% confidence interval. Adjusted for cervical dilatation at start of oxytocin infusion, fetal birth weight and epidural analgesia.

1587x1190mm (96 x 96 DPI)

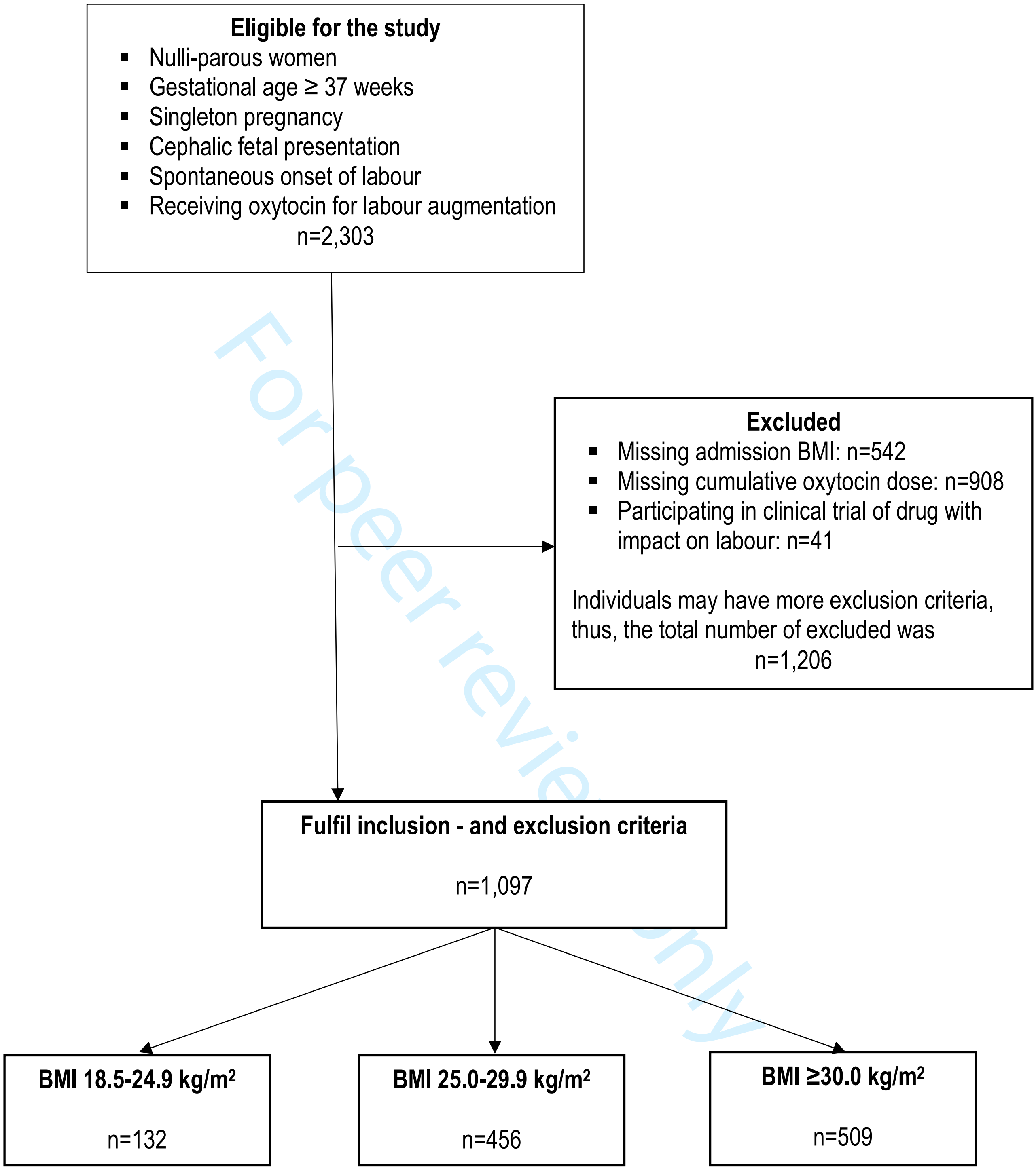

Figure S1. Flow chart of the study population

For peer review only

Table S1. Comparison of maternal, obstetric and neonatal characteristics in nulliparous women with singleton, term pregnancy with cephalic presentation, spontaneous start of labour and treatment with oxytocin infusion for labour augmentation included in the study and those excluded from the study.

	Included n=1,097	Excluded n=1,206	p-value
Maternal characteristics			
Maternal age (years)	28.5 [4.3]	28.5 [4.6]	NS*
Smoking during pregnancy (Yes/No)	35 / 1022 (3.3 / 96.7)	34 / 1091 (3.0 / 97.0)	NS†
Diabetes Mellitus (Yes/No)	12 / 2085 (1.1 / 98.9)	21 / 1185 (1.7 / 98.3)	NS†
Hypertension disorder (including Preeclampsia) (Yes/No)	31/1066 (2.8/97.2)	40 / 1166 (3.3 / 96.7)	NS†
Maternal weight in first trimester (kg)	68.9 [14.1]	68.1 [13.6], n=1,144	NS*
Gestational weight gain (kg)	15.1 [5.8]	14.8 [5.9], n=675	NS*
BMI in first trimester (kg/m^2)	24.8 [4.7]	24.7 [4.6], n=1,139	NS*
BMI at admission in labour (kg/m^2)	30.3 [5.0]	30.3 [5.0], n=664	NS*
Obstetric characteristics			
Gestational age at delivery (days)	282 [7.6]	282 [7.7]	NS*
Mode of delivery:			
Normal vaginal delivery	891 (81.2)	924 (76.6)	<0.01‡
Instrumental vaginal delivery	142 (12.9)	176 (14.6)	
Caesarean section (CS)	64 (5.8)	106 (8.8)	
Obstetric anal sphincter injury (Yes/No)	58 / 1039 (5.3 / 94.7)	49 / 1157 (4.1 / 95.9)	NS†
Indication CS (asphyxia/dystocia)	18 / 46 (28.1 / 71.9)	41 / 65 (38.7 / 61.3)	NS†
Epidural analgesia (Yes/No)	866 / 231 (78.9 / 21.1)	918 / 288 (76.1 / 23.9)	NS†
Postpartum haemorrhage (mL)	470 [368]	513 [406]	<0.001#
Postpartum haemorrhage >1000mL (Yes/No)	81 / 1008 (7.4 / 92.6)	98 / 866 (10.2 / 89.8)	0.03†
Neonatal characteristics			
Fetal birth weight (gram)	3560 [449]	3545 [466]	NS*
Apgar<7 at 5 minutes (Yes/No)	19 / 1078 (1.7 / 98.3)	32 / 1174 /2.7 / 97.3)	NS†
Apgar <4 at 5 minutes (Yes/No)	1 / 1096 (0.1 / 99.9)	4 / 1202 (0.3 / 99.7)	NS†
Umbilical arterial pH <7.0 (Yes/No)	14 / 1083 (1.3 / 98.7)	17 / 1189 (1.4 / 98.6)	NS†

Figures denote mean and [one standard deviation] or number and (%).
*One-way ANOVA; #Mann-Whitney U test; † Pearson's Chi-squared test (df=1). ‡ Pearson's Chi-squared test (df=2).
Level of significance p<0.05, NS= Not Significant

STROBE 2007 (v4) checklist of items to be included in reports of observational studies in epidemiology*
Checklist for cohort, case-control, and cross-sectional studies (combined)

Section/Topic	Item #	Recommendation	Reported on page #
**Title and abstract**	1	(*a*) Indicate the study's design with a commonly used term in the title or the abstract	1,2
		(*b*) Provide in the abstract an informative and balanced summary of what was done and what was found	2
**Introduction**			
Background/rationale	2	Explain the scientific background and rationale for the investigation being reported	4
Objectives	3	State specific objectives, including any pre-specified hypotheses	4
**Methods**			
Study design	4	Present key elements of study design early in the paper	5,6
Setting	5	Describe the setting, locations, and relevant dates, including periods of recruitment, exposure, follow-up, and data collection	5,6
Participants	6	(*a*) *Cohort study*—Give the eligibility criteria, and the sources and methods of selection of participants. Describe methods of follow-up<br>*Case-control study*—Give the eligibility criteria, and the sources and methods of case ascertainment and control selection. Give the rationale for the choice of cases and controls<br>*Cross-sectional study*—Give the eligibility criteria, and the sources and methods of selection of participants	5
		(*b*) *Cohort study*—For matched studies, give matching criteria and number of exposed and unexposed<br>*Case-control study*—For matched studies, give matching criteria and the number of controls per case	
Variables	7	Clearly define all outcomes, exposures, predictors, potential confounders, and effect modifiers. Give diagnostic criteria, if applicable	6,7
Data sources/ measurement	8*	For each variable of interest, give sources of data and details of methods of assessment (measurement). Describe comparability of assessment methods if there is more than one group	*6,7*
Bias	9	Describe any efforts to address potential sources of bias	5,6,7
Study size	10	Explain how the study size was arrived at	7
Quantitative variables	11	Explain how quantitative variables were handled in the analyses. If applicable, describe which groupings were chosen and why	6,7
Statistical methods	12	(*a*) Describe all statistical methods, including those used to control for confounding	7
		(*b*) Describe any methods used to examine subgroups and interactions	7
		(*c*) Explain how missing data were addressed	Table 1
		(*d*) *Cohort study*—If applicable, explain how loss to follow-up was addressed<br>*Case-control study*—If applicable, explain how matching of cases and controls was addressed	

For peer review only

		Cross-sectional study—If applicable, describe analytical methods taking account of sampling strategy	
		(*e*) Describe any sensitivity analyses	
**Results**			
Participants	13*	(a) Report numbers of individuals at each stage of study—eg numbers potentially eligible, examined for eligibility, confirmed eligible, included in the study, completing follow-up, and analysed	8, table 1
		(b) Give reasons for non-participation at each stage	5, Suppl Figure S1
		(c) Consider use of a flow diagram	Suppl Figure S1
Descriptive data	14*	(a) Give characteristics of study participants (eg demographic, clinical, social) and information on exposures and potential confounders	8,9 Table 1
		(b) Indicate number of participants with missing data for each variable of interest	Table 1
		(c) *Cohort study*—Summarise follow-up time (eg, average and total amount)	
Outcome data	15*	*Cohort study*—Report numbers of outcome events or summary measures over time	8,9 Table 1
		Case-control study—Report numbers in each exposure category, or summary measures of exposure	
		Cross-sectional study—Report numbers of outcome events or summary measures	
Main results	16	(*a*) Give unadjusted estimates and, if applicable, confounder-adjusted estimates and their precision (eg, 95% confidence interval). Make clear which confounders were adjusted for and why they were included	8,9
		(*b*) Report category boundaries when continuous variables were categorized	6
		(*c*) If relevant, consider translating estimates of relative risk into absolute risk for a meaningful time period	
Other analyses	17	Report other analyses done—eg analyses of subgroups and interactions, and sensitivity analyses	9
**Discussion**			
Key results	18	Summarise key results with reference to study objectives	10,13
Limitations	19	Discuss limitations of the study, taking into account sources of potential bias or imprecision. Discuss both direction and magnitude of any potential bias	10,11,12
Interpretation	20	Give a cautious overall interpretation of results considering objectives, limitations, multiplicity of analyses, results from similar studies, and other relevant evidence	12,13
Generalisability	21	Discuss the generalisability (external validity) of the study results	11
**Other information**			
Funding	22	Give the source of funding and the role of the funders for the present study and, if applicable, for the original study on which the present article is based	7,14

*Give information separately for cases and controls in case-control studies and, if applicable, for exposed and unexposed groups in cohort and cross-sectional studies.

Note: An Explanation and Elaboration article discusses each checklist item and gives methodological background and published examples of transparent reporting. The STROBE checklist is best used in conjunction with this article (freely available on the Web sites of PLoS Medicine at http://www.plosmedicine.org/, Annals of Internal Medicine at http://www.annals.org/, and Epidemiology at http://www.epidem.com/). Information on the STROBE Initiative is available at www.strobe-statement.org.

For peer review only
